# A Novel Interaction of Translocator Protein 18 kDa (TSPO) with NADPH Oxidase in Microglia

**DOI:** 10.1007/s12035-020-02042-w

**Published:** 2020-08-02

**Authors:** Meredith K. Loth, Sara R. Guariglia, Diane B. Re, Juan Perez, Vanessa Nunes de Paiva, Jennifer L. Dziedzic, Jeremy W. Chambers, Diana J. Azzam, Tomás R. Guilarte

**Affiliations:** 1 Department of Environmental Health Sciences, Mailman School of Public Health, Columbia University, New York, NY USA; 2 Department of Environmental Health Sciences, Robert Stempel College of Public Health & Social Work, Florida International University, Miami, FL 33199 USA

**Keywords:** TSPO, NOX2, Microglia, Heme, ROS, Redox homeostasis

## Abstract

In the brain neuropil, translocator protein 18 kDa (TSPO) is a stress response protein that is upregulated in microglia and astrocytes in diverse central nervous system pathologies. TSPO is widely used as a biomarker of neuroinflammation in preclinical and clinical neuroimaging studies. However, there is a paucity of knowledge on the function(s) of TSPO in glial cells. In this study, we explored a putative interaction between TSPO and NADPH oxidase 2 (NOX2) in microglia. We found that TSPO associates with gp91^phox^ and p22^phox^, the principal subunits of NOX2 in primary murine microglia. The association of TSPO with gp91^phox^ and p22^phox^ was observed using co-immunoprecipitation, confocal immunofluorescence imaging, and proximity ligation assay. We found that besides gp91^phox^ and p22^phox^, voltage-dependent anion channel (VDAC) also co-immunoprecipitated with TSPO consistent with previous reports. When we compared lipopolysaccharide (LPS) stimulated microglia to vehicle control, we found that a lower amount of gp91^phox^ and p22^phox^ protein co-immunoprecipitated with TSPO suggesting a disruption of the TSPO-NOX2 subunits association. TSPO immuno-gold electron microscopy confirmed that TSPO is present in the outer mitochondrial membrane but it is also found in the endoplasmic reticulum (ER), mitochondria-associated ER membrane (MAM), and in the plasma membrane. TSPO localization at the MAM may represent a subcellular site where TSPO interacts with gp91^phox^ and p22^phox^ since the MAM is a point of communication between outer mitochondria membrane proteins (TSPO) and ER proteins (gp91^phox^ and p22^phox^) where they mature and form the cytochrome b_558_ (Cytb_558_) heterodimer. We also found that an acute burst of reactive oxygen species (ROS) increased TSPO levels on the surface of microglia and this effect was abrogated by a ROS scavenger. These results suggest that ROS production may alter the subcellular distribution of TSPO. Collectively, our findings suggest that in microglia, TSPO is associated with the major NOX2 subunits gp91^phox^ and p22^phox^. We hypothesize that this interaction may regulate Cytb_558_ formation and modulate NOX2 levels, ROS production, and redox homeostasis in microglia.

**Electronic supplementary material:**

The online version of this article (10.1007/s12035-020-02042-w) contains supplementary material, which is available to authorized users.

## Introduction

Translocator protein 18 kDa (TSPO) is a widely used preclinical and clinical biomarker of brain injury and neuroinflammation that is able to detect diverse brain pathologies [1–3]. The cellular framework for using TSPO as a biomarker is based on the activation of microglia and astrocytes, the glial cell types that express and upregulate TSPO levels following nervous system insults and neuroinflammation [1, 3, 4]. TSPO expression is nearly undetectable in the normal brain neuropil but increases markedly and selectively at primary and secondary sites of brain injury and neuroinflammation [1, 3]. More specifically, TSPO has been used as a sensitive biomarker of reactive gliosis and inflammation associated with a variety of brain insults including chemical-induced neurotoxicity [5–10], ischemia [11], traumatic brain injury [12, 13], and a number of neurodegenerative disorders with an inflammatory component such as Alzheimer’s disease, Parkinson’s disease, amyotrophic lateral sclerosis (ALS), multiple sclerosis, and virus-induced neuroinflammation [14–18]. In this way, TSPO can be used to evaluate the neurotoxic potential of chemicals and to track active neuroinflammation and brain injury in a multitude of neurological and neurodegenerative conditions in humans using positron emission tomography imaging [1, 3].

Despite the extensive use of TSPO in preclinical and clinical neuroimaging studies to detect glial cell activation as a result of brain injury and neurodegeneration, the function of TSPO in glial cells has received limited attention, and studies on the function of TSPO in primary microglia or astrocytes are lacking. During the last several decades, a number of diverse functions have been attributed to TSPO, including cholesterol transport into the mitochondria for steroidogenesis; regulation of the mitochondrial permeability transition pore (mPTP); reactive oxygen species (ROS) production; and porphyrin/heme transport, among several other putative functions [1, 19–22]. Most of these studies were performed in steroidogenic primary cultures or cell lines, with limited studies in primary glial cells. Further, the first effort to generate global TSPO knockout (KO) mice described that the TSPO-KO mice were unable to survive and that global *Tspo* deletion was embryonic lethal [21]. However, new evidence has emerged that has questioned the dogma that global deletion of *Tspo* is embryonic lethal and that TSPO plays an essential role in steroidogenesis or regulates the mPTP. Since 2014, studies have shown that global TSPO-KO mice are viable and that TSPO does not appear to be required for steroidogenesis [23–25] or to participate in the regulation of the mPTP [26]. Structural biology studies also show that oligomerization of TSPO is unlikely to form a pore for cholesterol transport, as previously proposed [27]. These contradictory findings have generated significant controversy and confusion in the scientific literature related to the cellular function(s) of TSPO. Therefore, elucidating the functional significance of TSPO upregulation in glial cells under conditions of diverse neuropathology is important in order to advance the understanding of TSPO and glial cell biology.

Microglia are the resident immune cells of the brain with an ability to sense and respond to cellular signals resulting from disruption of brain homeostasis [28, 29]. Microglia have a specific morphological and functional response when they are activated, changing from a ramified morphology to one with thickened and shortened processes [28, 29]. Additionally, microglia significantly increase TSPO expression when they are activated with a time course that is dependent on the type and degree of injury [1, 3]. Although TSPO appears to play a role in the neuroinflammatory response, there is a lack of knowledge concerning the precise molecular and cellular function(s) of TSPO in the microglial response to brain injury.

Our laboratory has previously shown that microglia exposed to physiologically relevant concentrations (1–100 nM) of TSPO-specific ligands (i.e., (R)-PK11195 and Ro5-4864) increased ROS production that was abrogated by different types of NADPH oxidase (NOX2) inhibitors [30]. These experiments provided the initial evidence of a putative association between TSPO and NOX2 in microglia. NOX2 is a major source of ROS production in the central nervous system, and similar to TSPO, it is highly enriched in microglia [31, 32]. NOX2 is a multi-subunit enzyme composed of the cytosolic subunits p40^phox^, p47^phox^, p67^phox^, the small G protein Rac1, and the integral membrane subunits p22^phox^ and gp91^phox^ (gp91^phox^ is also called NOX2). The principal membrane subunit gp91^phox^ is processed and matured in the endoplasmic reticulum (ER) via the incorporation of two heme molecules into its glycosylated precursor gp65, followed by dimerization with p22^phox^ to form the heterodimer, flavocytochrome b_558_ (Cytb_558_), and further glycosylation in the Golgi [33–36]. The Cytb_558_ heterodimer then traffics to the plasma membrane and endosomal compartments. In the plasma membrane, Cytb_558_ contributes the catalytic core of the membrane-embedded NOX2 enzyme [33–36]. Importantly, the incorporation of heme into gp91^phox^ in the ER is essential for Cytb_558_ formation, since in the absence of heme, gp91^phox^ and p22^phox^ do not form a dimer (i.e., Cytb_558_) and are degraded by the proteasome [33–36].

In phagocytes, activation of NOX2 occurs when a stimulus promotes the phosphorylation of the cytosolic subunit p47^phox^. The cytosolic subunits subsequently translocate to the membrane where they assemble with Cytb_558_ to form a membrane-bound enzymatic complex to generate superoxide by the transfer of two electrons from NADPH to molecular oxygen [37]. Studies have shown that cholesterol is also needed for the NOX2 cytosolic subunits to translocate to the plasma membrane or lipid rafts in order to form an active NOX2 complex [38, 39].

In this study, we provide new experimental evidence of an association between TSPO and the NOX2 subunits gp91^phox^ and p22^phox^ in microglia. We provide a working model suggesting that this interaction may be associated with the TSPO-mediated transfer of heme from mitochondria to gp91^phox^ in the ER. Thus, TSPO may regulate NOX2 levels, ROS production, and redox homeostasis in microglia.

## Materials and Methods

### Culture of Cell Lines

Human female HEK293T (RRID: CVCL_0063) and mouse female macrophage J774.A1 (RRID: CVCL_0358) (provided in 2011 by Dr. Martin Pomper’s laboratory, Johns Hopkins Medical Institution, Baltimore, MD) cell lines were maintained in DMEM and RPMI media (Life Technologies, Carslbad, CA) respectively, with 10% fetal bovine serum (FBS; Hyclone, GE Healthcare Life Sciences, Marlborough, MA) and 1% (vol/vol) penicillin-streptomycin. These cell lines were used to validate the quality of the antibodies used in the microglia experiments and not for studies on the TSPO-NOX2 subunit interaction.

### Primary Microglia Cell Culture

All studies examining the TSPO-NOX2 subunit association were performed in murine primary microglia cultures. Primary murine mixed glial cell cultures were prepared using a modified version of the glial culture technique as previously described using post-natal day (PN) 1-3 C57/Bl6 mouse pups [40, 41]. Breeder animals were obtained from Envigo (Indianapolis, IN) and bred in house (1 male, 1 female) to generate litters until female breeders were 9 months of age. For each dissection, 10–18 brains (dependent on litter number and size) were extracted and meninges were removed from PN1-3 C576/Bl6 mouse pups. Brain tissue was dissociated with 0.25% trypsin (Thermo Fisher Scientific, Waltham, MA), in a 37 °C water bath for 30 min, and tissue was shaken by hand every 5 min. Trypsinization was stopped with equal volume of DMEM F-12 media (Life Technologies, Carslbad, CA) supplemented with 10% FBS (Hyclone, GE Healthcare Life Sciences, Marlborough, MA) and 1% (vol/vol) penicillin-streptomycin (Life Technologies, Carlsbad, CA). Tissue was triturated using various sized pipettes to create a single cell suspension of brain tissue and ultimately filtered using a 70-μm mesh filter (BD, Franklin Lakes, NJ). Single cell suspension was combined with fresh DMEM F-12 media such that cells were seeded with 1 mouse brain across 2 T75 flasks (Corning, Corning, NY) with each flask containing a final volume of 10 mL. Cultures were maintained in a humidified incubator at 37 °C with 95% air/5% CO_2_. Media was changed at day 4–5 post dissection. After 12–14 days in culture, microglia were separated from the glial cultures by shaking the flasks for 3 h at 120 r.p.m. in a temperature-controlled shaking incubator and collecting the floating cells in the media. After centrifugation for 10 min at 400×*g*, cell viability was determined by trypan blue exclusion, and cells were plated at various densities according to the experiment being performed (12 well plate: 1 × 10^5^ cells per well on poly-l-lysine coated coverslips (Fisher Scientific, Hampton, NH. 60-cm plate: 5 × 10^5^ cells per plate). Approximately 94% or greater of the adherent cells were positive for the microglia-specific marker Mac-1 as determined by immunostaining. Cells rested overnight (16–20 h) before any dosing or assays were performed. Animals used for these studies were in accordance with relevant guidelines and regulations, and all studies were reviewed and approved by Columbia University and Florida International University Animal Care & Use Committees.

### Antibody Validation Using siRNA Transfection

HEK293T cells were transfected with siRNAs using Lipofectamine 2000 (Life Technologies, Carlsbad, CA) and harvested at 48-h post-transfection. The Silencer Select Validated siRNA (ID: s224728, Applied Biosystems-Thermo Fisher Scientific, Waltham, MA) was used to knockdown TSPO in HEK293T cells, with the Silencer Select Negative Control #1 siRNA (Applied Biosystems-Thermo Fisher Scientific, Waltham, MA) as scramble siRNA control.

### Antibody Validation Via Gene Silencing by Spinoculation Using shRNA Lentiviral Particles

Due to the sensitive nature of primary microglia to be activated by manipulation and their inability to be cultured for long periods of time, validation of TSPO, gp91^phox^, and p22^phox^ antibodies was performed using shRNA transduction in J774.A1 murine macrophage cell line. The J774.A1 macrophages cell line is of the same species as the primary microglia cultures (mouse) and a related cell type as microglia are considered macrophages of the central nervous system. All shRNAs and empty vectors used in this study were from Sigma-Aldrich MISSION (St. Louis, MO). Clones tested included TSPO (*Tspo*): TRCN00000102106 (#102106); TRCN00000102107 (#102107); TRCN00000102109 (#102109). gp91^phox^(*Cybb*): TRCN00000435339 (#435339); TRCN00000422819 (#422819); TRCN00000240564 (#240564). p22^phox^ (*Cyba*): TRCN00000240562 (#240562); TRCN00000240565 (#240565); TRCN00000011889 (#011889). Clones #102106 (TSPO), #435339 (gp91^phox^), and #240565 (p22^phox^). A suspension of 100,000 cells/ml was treated with 8 μg/ml of hexadimethrine bromide (107,689, Sigma-Aldrich, St. Louis, MO) and lentiviral particles at a multiplicity of infection (MOI) of 15. Cells were centrifuged at 800×*g* for 30 min at room temperature and were resuspended and plated at a density of 20,000 cells/well. After 48 h, cells were treated with 1 μg/ml puromycin (A11138, Life Technologies, Carlsbad, CA) for 4.5 days to select for cells which had been transduced. After puromycin selection, cells were grown in fresh media for 7 days before harvesting for Western blot.

### LPS Treatment

Bacterial lipopolysaccharide (LPS, Sigma-Aldrich, St. Louis, MO) was dissolved in phenol red free DMEM F-12 media (Life Technologies, Carlsbad, CA) to create a stock solution of 1 mg/mL. A solution of 100 ng/mL of LPS was made fresh for each experimental exposure. J774.A1 cells were exposed to 1 μg/ml for 3 and 24 h. Microglia were exposed to 100 ng/mL of LPS for time of exposure indicated.

### Western Blotting

Standard Western blotting as seen in Supplemental Figure S1 was performed using the anti-TSPO antibody (Epitomics/Abcam, Cambridge, UK, 3623-1, 1:1000; RRID:AB_10897701) and anti-ß-actin antibody (Santa Cruz, Santa Cruz, CA, sc-1616, RRID:AB_630836, 1:2500) as the primary antibodies. The Odyssey Infrared Imaging system was used for fluorescence immunoblotting with IRDye dye-labeled secondary antibodies (LI-COR Biosciences, Lincoln, NE, 1:5000) For Western blotting methods used in immunoprecipitation experiments, see below.

### [^3^H]-R-PK11195 Binding Assay

Binding assays were performed using macrophage J774.A1 cells harvested 3 h or 24 h after treatment with vehicle or LPS. On the day of the assay, cell pellets were thawed and sonicated in 50 mM Tris-HCl assay buffer (pH 7.4) and distributed into assay tubes to provide approximately 25–150-μg protein/tube. Protein concentration was determined by Bradford protein assay using bovine serum albumin as a standard. The final assay volume was 0.5 ml and all tubes were kept on ice during preparation. Cell suspensions were incubated in triplicate and binding was determined using [^3^H]-R-PK11195 (specific activity 84.8 Ci/mmol, Perkin Elmer, Waltham, MA) at a 2–2.5 nM concentration. Nonspecific binding was measured in the presence of 50 μM non-radioactive PK11195 (Sigma-Aldrich, St. Louis, MO). Assay tubes were incubated for 1 h at 4 °C. The reaction was terminated by filtration through Whatman GF/B filter paper (Brandel, Gaithersburg, MD) with a Brandel harvester system (Brandel, Gaithersburg, MD). Filters were washed three times with 4 ml of ice-cold assay buffer. Radioactivity retained in the filters was measured by liquid scintillation spectrometry using 10 ml of complete counting cocktail (Econo-Safe; Research Products International).

### Transgenic Mice

The animal procedures described in this study were approved by Florida International University Institution Animal Care & Use Committee. Six mice (4 female and 2 male) heterozygous for TSPO were obtained from Helmholtz Zentrum Munich German Mouse Clinic as part of the International Mouse Phenotyping Consortium (IMPC) and INFRAFRONTIER/European Mouse Mutant Archive (EMMA). TSPO tm1b mice (RRID:IMSR_EM:09345) were produced on a C57BL/6NTac background and mice were produced by treating 2-cell embryos with Cre enzyme as described previously [42]. Tm1b allele embryos have a reporter-tagged deletion allele (post-Cre). Critical exons, in this case exons 2 and 3 of *Tspo*, are deleted by creating a frame shift using the Cre method. Genotypes of animals were validated through endpoint PCR. When TSPO heterozygous mice arrived in the USA, animals were quarantined for 2 weeks and then allowed to sexually mature until all animals were at least 8 weeks of age. To generate TSPO wildtype, heterozygous, and knockout animals, TSPO heterozygous animals were bred in harem (2 females, 1 male), and used as breeders until the female was 8 months of age or the female stopped producing litters after 3 attempts at breeding.

### Immunoprecipitation and Western Blots

Cells were plated onto 60-mm dishes at a density of 500,000 cells per dish. Cells were harvested in a mild lysis buffer containing 0.2 mM sodium orthovanadate, 5 mM sodium fluoride, 0.5% NP-40, 2.5 mM ethylenediaminetetraacetic acid (EDTA), 150 mM potassium chloride, and 10 mM Tris, pH 7.4 [43]. Multiple microglia extractions were combined in order to achieve a 415 μg protein concentration per treatment condition for each immunoprecipitation (IP) experiment. Cells were harvested for whole cell protein levels using the method of [44]. Protein concentration was determined using the Lowry assay.

A sample of the initial cell lysate was retained for analysis of total protein and Western blot and it is referred to as the “input fraction.” For each immunoprecipitation experiment, cell lysates were pre-cleared by incubating the lysate with 50 μL of IgG coated beads (Protein G Dynabeads) (Life Technologies, 10-007-D, Carlsbad, CA) for 2 h to eliminate any nonspecific binding to beads. Samples were then incubated with the anti-TSPO antibody (Abcam, Cambridge, MA, ab109497, RRID: AB_10862345, 1:10 (μg/μL)) overnight at 4 °C. Next, lysates were incubated for 2 h with 200 μL of Protein G Dynabeads to allow the F_c_ region of the antibody to bind to the IgG coated beads. All IP-related incubations were performed at 4 °C. An IP sample without any TSPO antibody was run in parallel as a negative control for the IP (no primary pulldown) to ensure specificity of the bands detected. Proteins were separated on 4–15% TGX Precast Gels (Biorad, Hercules, CA) and transferred to polyvinylidene difluoride (PVDF) membranes. For non-IP samples (i.e., the input fraction), 25 μg of protein was loaded per condition per lane. Immobilon-FL PVDF Western blot membranes (EMD Millipore, Burlington, MA, IPFL00010) were incubated with corresponding primary antibodies: goat anti-TSPO (Abcam, Cambridge, MA, ab118913, RRID:AB_10898989 1:1000); rabbit anti-TSPO (Abcam, Cambridge, MA, 109497, RRID:AB_10862345 1:1000); mouse anti-gp91^phox^ (BD Biosciences, San Jose, CA, 611415, RRID:AB_398937, 1:200); mouse anti-p22^phox^ (Santa Cruz Biotechnology, Dallas, TX, sc-130,551, RRID:AB_2245805, 1:100); rabbit anti-mouse VDAC (Abcam, Cambridge, MA, ab15895, RRID:AB_2214787, 1:500). Quick Western Kit IRDye 680RD (LI-COR Biosciences, Lincoln, NE, 926-69100) was used as it does not bind to denatured mouse or rabbit monoclonal antibodies. Standard Western blot LI-COR (Lincoln, NE) secondary antibodies (IRDye 800 CW Donkey anti-Mouse and IRDye 680LT donkey anti-rabbit, 1:10,000) were also used to confirm the specificity of Quick Western Kit IRDye 680RD (LI-COR Biosciences, Lincoln, NE, 926-68100).

### Gene Expression and RT-PCR

Gene expression for *Tspo*, *Cybb*, *Cyba*, *VDAC1*, and *GAPDH* was performed. RNA was isolated from primary mouse microglial cells treated with vehicle or 100 ng/mL LPS for 18 h by using RNAqueous Micro Kit (AM1931, Invitrogen, Carlsbad, CA). RNA content was measured using NanoDrop spectrophotometer (Thermo Fisher Scientific, Waltham, MA). Reverse transcription of RNA was performed with High-Capacity RNA-to-cDNA kit (Applied Biosystems, Thermo Fisher Scientific, Waltham, MA). qRT-PCR was performed using 1 μl of cDNA diluted to 20 ng/μl, TaqMan multiplex master mix (Applied Biosystems, Thermo Fisher Scientific, Waltham, MA) and TaqMan mouse primers (Thermo Fisher Scientific, Waltham, MA) in a final reaction of volume of 10 μl. Primers used included Tspo (Mm00437828_m1-FAM-MGB); Cybb (Mm01287743_m1-FAM-MGB); Cyba (Mm00514478_m1-FAM-MGB); Vdac1 (Mm00834272_m1-FAM-MGB); and Gapdh (Mm999999915_g1-VIC-MGB). The results were evaluated using QuantStudio Real-Time PCR Software v1.3 (Thermo Fisher Scientific, Waltham, MA). Amplification specificity was confirmed by melting curve analysis, and the quantification was carried out using the ΔΔCt method [45]. All samples were normalized to Gapdh. Data from six independent experiments were run on the same plate for each gene. All individual samples were run in triplicate.

### Immunocytochemistry

Microglia (1 × 10^5^) were plated on poly-l-lysine glass coverslips (Fisher Scientific, Pittsburgh, PA, 08-774-383) in 12-well plates. Cells were treated for 18 h with vehicle (media) or 100 ng/mL LPS in media supplemented with 2% FBS (2 mL solution per well; Hyclone, GE Healthcare Life Sciences, Marlborough, MA). Immunocytochemistry was performed via conventional techniques. That is, cells were fixed with 4% paraformaldehyde (PFA) and permeabilized with 0.2% Triton X-100. Blocking was performed with 10% normal donkey serum (Jackson Laboratories, Bar Harbor, ME) for 3 h before being incubated with primary antibodies. Goat anti-TSPO (Abcam, Cambridge, MA, ab118913, RRID:AB_10898989, 1:500); mouse anti-gp91^phox^ (BD Biosciences, San Jose, CA, 611415, RRID:AB_398937, 1:100); mouse anti-p22^phox^ (Santa Cruz Biotechnology, Dallas, TX, sc-130,551, RRID:AB_2245805, 1:100); rabbit anti-VDAC (Abcam, Cambridge, MA, ab15895, RRID:AB_2214787, 1:500) were used. After washing, cells were incubated with appropriate Alexa Fluor secondary antibodies for 1 h (Life Technologies, Carlsbad, CA; AF 488, 594, and 647, 1:500). Coverslips were mounted with Prolong with DAPI (Life Technologies, Carlsbad, CA) to counterstain for cell nuclei.

### Immunohistochemistry

Free-floating brain sections from PFA-perfused mice were sectioned at 40 μm using a freezing microtome (Leica Microsystems Inc., Bannockburn, IL). Sections were stored at − 20 °C in cryoprotectant consisting of 50% glycerol in 0.05 M phosphate buffer. For immunofluorescence triple labeling of Mac-1, TSPO, and gp91^phox^, free-floating brain sections were washed with 1X Tris-buffered saline (TBS) for 60 min, with fresh TBS every 10 min. Sections were then blocked with 5% normal donkey serum (Jackson Laboratories, Bar Harbor, ME) containing 0.2% Triton X-100 for 1 h followed by primary antibody incubation: rat-anti-Mac-1 (BD Biosciences, San Jose, CA, 553308, 1:250, RRID:AB_394772), rabbit anti-TSPO (Abcam, Cambridge, MA; ab109497, RRID:AB_10862345, 1:500), and goat anti-gp91^phox^ (Santa Cruz Biotechnology, Dallas, TX, (sc-5827), RRID:AB_647636, 1:150) at 4 °C overnight in 2 mL of solution. After washing, sections were incubated with appropriate Alexa Fluor secondary antibodies (Life Technologies, Carlsbad, CA; 1:500) for 1 h and underwent another series of washes post-secondary antibody incubation. Sections were mounted on slides and coverslipped with Prolong Gold Antifade Mountant (Life Technologies, Carlsbad, CA) to preserve signal intensity and brightness.

### Immunofluorescence Imaging and Analysis For In Vitro Experiments

Immunofluorescence-labeled cells were imaged at × 60 magnification with a 1.6× zoom using a laser scanning confocal microscope (Fluoview FV10i, Olympus, Center Valley, PA), utilizing the FV10 image software. All coverslips stained under the same conditions were imaged using the same scanning parameters on the same day. Images were scanned at a resolution of 512 × 512 pixels. Five to six confocal stacks were obtained for each experimental condition with at least 25 cells counted per condition. In order for a cell to be counted, the entire cell needed to be within the frame of the image. Confocal stacks were projected as single images using the maximum fluorescence FV10 software. Maximum intensity projection images for each channel were converted to a gray scale image using Photoshop.

All images were analyzed using Metamorph Offline (Molecular Devices, Downington, PA). Threshold values were determined by eye by a single experimenter for all experiments, and threshold values were kept consistent across both vehicle and LPS conditions within an experiment. Threshold values and colocalization results were cross-checked by having a second experimenter perform colocalization analysis on a subset of cells and compare results of the 2 experimenters; results of the 2 experimenters varied by < 2%. Regions of interest (ROIs) were manually created around each cell in the VDAC channel. ROIs were drawn to be the shape of the cell, with about a 10% buffer of space between the ROI outline and the signal from the cell. ROIs were transferred to the TSPO channel and the NOX2 subunit channel using the Metamorph Transfer Function such that each ROI outlined each cell, in each channel. ROIs were cross-checked in the TSPO and NOX2 subunit channels to ensure all fluorescent signal due to cell expression was captured within the ROI for each channel.

For colocalization analyses, gray scale images of each wavelength (each protein) were used to examine the area of colocalized pixels of both wavelengths to calculate the percent colocalization of their signals using the Metamorph Application “Measure Colocalization.” Percent colocalization was calculated as previously described [46]. Briefly, colocalization of protein A with protein B equals area (A with B) divided by total area (A) where A and B are individual wavelengths for the same image. To assess colocalization of 3 proteins (proteins A, B, and C), a new image (AB’) was generated that consisted of pixels when A and B colocalized. The colocalization of proteins (A + B) with C was then quantified using images AB’ and C.

### Immunofluorescence Imaging and Analysis For Ex Vivo Experiments

Four to six images were acquired in the ventral posteromedial thalamic nuclei from horizontal brain sections in age matched wildtype and Sandhoff disease animals with four male animals per group. This area of the thalamus was chosen based off of prior studies by Loth et al. [47] that have shown TSPO upregulation specific to Sandhoff disease in this brain region. Imaging was done at × 60 magnification with a 1.6× zoom using a laser scanning confocal microscope (Fluoview FV10i, Olympus, Center Valley, PA), utilizing the FV10 image software. Colocalization was calculated as describe above for proteins A, B, and C to assess when TSPO colocalizes with gp91^phox^, what percentage of that colocalizes with the microglia marker Mac-1. Colocalization analysis was performed on the entire image (no ROIs were drawn) as expression of all markers was consistent across the field of view in the image.

### Duolink Proximity Ligation Assay

Briefly, samples were incubated with primary antibodies to bind to the two proteins of interest. Secondary antibodies conjugated with positive and negative oligonucleotides (proximity ligation assay (PLA) probes) were then incubated with the cells. Next, ligase and two additional oligonucleotides were introduced, and these oligonucleotides hybridize to the PLA probes, and if in close enough proximity (~ 30–40 nm), form a closed circle. In the DNA circle, one of the antibody conjugated DNA probes serves as a primer for rolling circle amplification (RCA) and a repeated sequence (concatemeric) product was generated when DNA polymerase and nucleotides were added. Fluorescently labeled oligonucleotides proceed to bind to the RCA product and allowed visualization of the protein-protein interaction as single dots via fluorescence microscopy. PLA experiments were performed according to the manufacturer’s instructions with minor modifications (Sigma-Aldrich, St. Louis, MO; Duolink In Situ Detection Reagents Red, DUO92008) including increase volume of solution for each coverslip. After vehicle or LPS treatment, cells were fixed with 4% PFA. Cells were permeabilized with 0.2% Triton X-100 for 25 min and blocked with 10% normal donkey serum before being incubated for corresponding primary antibodies for 72 h at 4 °C: goat anti-TSPO (Abcam, Cambridge, MA, ab118913, RRID:AB_10898989, 1:500); mouse anti-gp91^phox^ (BD Biosciences, San Jose, CA, 611415, RRID:AB_398937, 1:200); mouse anti-p22^phox^ (Santa Cruz Biotechnology, Dallas, TX, sc-130,551, RRID:AB_2245805, 1:100); rabbit anti-mouse VDAC (Abcam, Cambridge, MA, ab15895, RRID:AB_10862345, 1:500) with 100 μL of solution per coverslip. After washing, cells underwent exposure to PLA Probes (Goat PLUS RRID:AB_10971336, and Mouse MINUS RRID:AB_2713942; or Goat PLUS RRID:AB_10971336, and Rabbit MINUS, RRID:AB_2810942); ligation solution, and amplification solution as per manufacturer’s instructions.

### TSPO Immuno-Gold Electron Microscopy in Primary Microglia

Microglia were plated at a density of 5 × 10^5^ in a 6-cm plate. Cells were treated with either vehicle or LPS (100 ng/mL; 3 ml of solution per plate) for 18 h. Following exposure, cell culture media was removed from the cells and replaced with a 4% PFA and 0.1% glutaraldehyde fixative. The fixative was left on the cells for 1 min and replaced with two successive changes of new media. Cells were left to fix for a total of 30 min. After fixation, a 1% sodium borohydride solution for antigen retrieval was used to rinse the cells for 20 min. After antigen retrieval, cells were cryoprotected using incubations of increasing concentrations of an 8% glycerol and 20% sucrose solution dissolved in 0.1 M phosphate-buffered saline (PBS) before placement into the − 80 °C freezer. The freezing served to rupture the cell membranes less harshly than traditional detergents. Cryoprotectant was removed from the cells, using successively increasing concentrations of PBS in PBS-cryoprotectant mixture. The cryoprotection and freezing process was repeated twice to ensure antibody penetration into the fixed cells. Nonspecific binding sites were then blocked in the cells using a 30-min incubation in a 5% BSA-PBS blocking buffer. After, cells were incubated in primary antibody for 24 h at room temperature followed by 48 h at 4 °C using a 1:200 dilution of monoclonal rabbit anti-TSPO antibody (Abcam, Cambridge, MA) in the 5% BSA blocking buffer. After incubation in the primary antibody, cells were rinsed four times in PBS and were left to incubate in a 1:200 diluted 0.5-nm gold nanoprobe secondary antibody in 5% BSA blocking buffer, for 24 h at room temperature followed by 48 h at 4 °C. After secondary antibody incubation, cells were rinsed four times in PBS and two times in a 245 mM sodium acetate buffer. Gold nanoprobes were then enhanced using a silver HQ kit (Nanoprobes, Yaphank, NY). The enhancement reagent was prepared using the kit directives and applied to the cells for a total of 4 min for silver enhancement. The silver enhancement reaction was halted using a 0.1 M sodium thiosulfate rinse for 3 min, followed by three quick sequential rinses with sodium acetate buffer and a rinse in 0.1 M PBS. Silver enhancement procedures were carried out in the dark, as light exposure could interfere with the reaction and result in the formation of insoluble precipitates. Following the completion of the steps for immunolabeling, cells were postfixed using a 2% glutaraldehyde solution for 10 min, rinsed in PBS, and incubated in 0.5% osmium tetroxide in PBS for lipid fixation. Cells were then dehydrated using stepwise increasing concentrations of ethanol in an ethanol-water dilution series. Once the cells reached incubation in 100% ethanol, they were gently scraped from the Petri dish and pipetted into a microcentrifuge tube containing propylene oxide. Cells were centrifuged at 1500 rpm in a microcentrifuge to keep the cells at the bottom of the tube. All traces of ethanol were removed from the cells using propylene oxide. Cells were infiltrated with resin using increasing concentrations of Spurr resin in a Spurr Resin-propylene oxide mixture (25%, 50%, 75%, 100%, 100%). Scraping at the 100% ethanol step in this multistep protocol helped to minimize cell loss. Moreover, incubations in osmium tetroxide, uranyl acetate, and silver increased the density of the cells, which facilitated fewer, low-speed centrifugation. This approach aided in the preservation of ultrastructure. After infiltration, 500 μL of fresh resin was used to replace old resin in the microcentrifuge tube. The resin polymerized over 24 h at 60 °C. After polymerization, the resin block was removed from the microcentrifuge tube using a razor blade. Sixty-nanometer ultrathin sections were cut using a Diatome diamond knife (EMS Diasum, Hatfield, PA) on Leica (Buffalo Grove, IL) Ultracut UCT ultramicrotome. Two hundred mesh nickel grids were used to support the sections. Sections were stained using an aqueous 4% uranyl acetate solution for 30 min at room temperature in the dark. Sections were rinsed in water and stained in a lead citrate solution for 6 min. Sections were rinsed in water and left to dry before examination in a FEI Tecnai Spirit Transmission Electron Microscope operated at 60 kV. Images were obtained using an AMT camera and image analysis software.

### Flow Cytometric Assays

To measure cellular ROS and surface TSPO expression levels, the CellROX® green flow cytometry assay kit was used (Thermo Fisher, Waltham, MA; C10492). Immediately after harvesting from a mixed glia culture, primary microglia cells were treated with and without 5000 μM *N*-acetyl cysteine (NAC), a ROS scavenger, for 1 h at 37 °C, followed by 400 μM of tert-butyl hydroperoxide (TBHP), an ROS inducer, for 45 min at 37 °C. Cells were then stained with 500 nM CellROX® Green Reagent for 45 min. Cells were also stained for surface TSPO expression without cellular permeabilization by adding TSPO-conjugated PE antibody (Abcam, Cambridge, MA; ab208836, 1:100) followed by fixation with 3% PFA. The CellROX® green flow cytometry kit was used in conjunction with SYTOX® Red Dead Cell Stain to differentiate live cells from dead cells. Cells were then analyzed by Becton Dickinson FACSMelody. Quantification of CellROX® green ROS probe signal was determined by the mean fluorescence intensity (MFI) using FlowJo software.

### Bioinformatic Model and Alignment of TSPO

Using established software sources, we aligned and modeled human TSPO (hTSPO) from the Uniprot sequence P30536-1 (accessed on May 30, 2020) to mouse TSPO (mTSPO; PDB: 2mgy.1) bound to PK11195 and *Rhodobacter sphaeroides* (rTSPO; PDB: 4uc1.1.B) bound to protoporphyrin IX (PPIX). Protein sequences for these structures were obtained through sequences deposited in the SWISS-MODEL workspace [48] accompanying the deposited structures listed above. The sequences were aligned to identify conserved sequences using the multiple alignment viewer, MView, produced by the European Bioinformatics Institute (EMBL-EBI) [49]. To model the hTSPO to the mTSPO PDB 2mgy.1 structure, we used the SWISS-MODEL software [48] and we compared the structure to other TSPO structures from mTSPO (PDB: 2n02.1) and rTSPO (4uc1.1, 4uc1.2, and 4uc3.1) to ensure the hTSPO model was in agreement with the major topological features of existing crystal structures. The hTSPO model was provided with a coloring scheme that permitted the distinct viewing of helices and other secondary structure elements, and surface topology predictions were used to interrogate the presence of hydrophobicity and aromaticity within the model. Next, both the primary sequence and structural model of hTSPO were entered in to the HemeBind software [50] to identify potential amino acids capable of interacting with heme. The residues were then examined and visualized on the hTSPO model using the SWISS-MODEL software package. Figure images were prepared by exporting renderings from the SWISS-MODEL software into Photoshop, where only cropping of white edges and labeling were performed. Otherwise, the image was unaltered after export from SWISS-MODEL.

### Statistical Analysis

Values are expressed as mean ± standard error of the mean (SEM). Student’s paired *t* tests were performed for Western Blot analyses and colocalization analyses. A two-way analysis of variance was performed for colocalization analysis between wildtype and Sandhoff mice at 4 different ages. For all tests, significance level was set at *p* < 0.05. For percent vehicle values, statistics were performed on log-transformed values.

## Results

### Co-immunoprecipitation of TSPO with gp91^phox^, p22^phox^, and VDAC in Microglia

To explore the putative association between TSPO and the NOX2 principal subunits gp91^phox^ and p22^phox^, we performed TSPO immunoprecipitation experiments of primary mouse microglia using antibodies independently validated in our lab (Supplementary Fig. S1) under both LPS-activated and vehicle conditions. Primary murine microglia cultures were greater than 94% pure as determined by macrophage integrin 1 (Mac-1 also called CD11b) immunostaining (Supplementary Fig. S2). For these studies, we also used voltage-dependent anion channel (VDAC) as a positive control because TSPO and VDAC have been shown to co-immunoprecipitate and colocalize in mitochondria [51, 52].

**Fig. 1:**
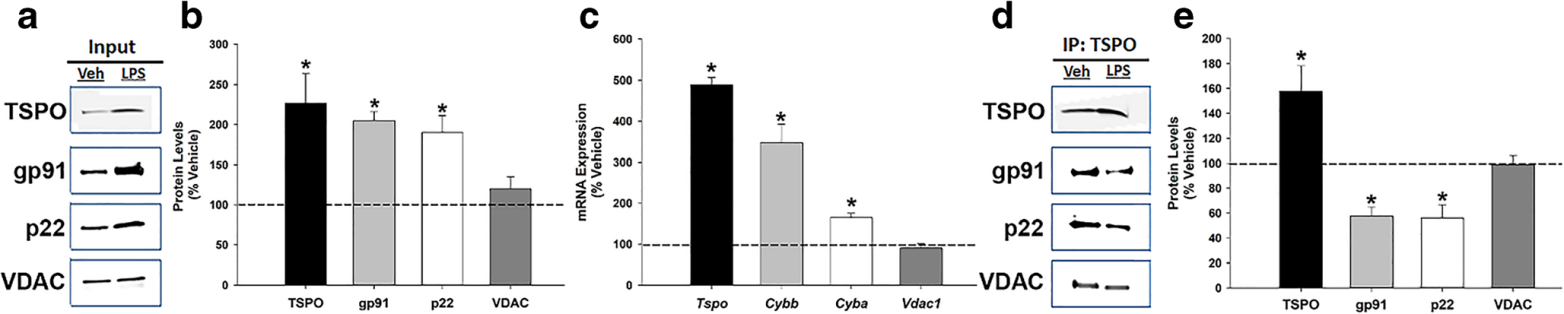
Co-immunoprecipitation of TSPO, gp91^phox^, p22^phox^, and VDAC and effect of LPS activation on protein-protein interaction in primary microglia. **a** Western blot of primary microglia whole cell extracts (input) after 18-h exposure to vehicle (media) or 100 ng/mL LPS. **b** TSPO, gp91^phox^, and p22^phox^ protein levels in whole cell extracts were significantly increased as a result of LPS stimulation, while VDAC protein levels did not change relative to vehicle condition (*n* = 7–8 independent experiments; TSPO: *p* = 0.001; gp91: *p* < 0.001; p22: *p* < 0.001; VDAC: *p* = 0.346). **c** Quantitative real-time PCR analysis of gene expression levels of the proteins analyzed in A in LPS-activated compared to non-activated microglia (*n* = 7 independent experiments; TSPO: *p* < 0.001; gp91: *p* = 0.002; p22: *p* < 0.001; VDAC: *p* = 0.382). **d** Western blot of the immunoprecipitation fraction (IP) with the TSPO antibody from vehicle and LPS-activated microglia confirms that gp91^phox^, p22^phox^, and VDAC proteins co-immunoprecipitates with TSPO. **e** Quantification of protein levels in the TSPO IP fraction from LPS-stimulated microglia shows increased TSPO protein compared to vehicle-treated microglia. On the other hand, the amount of gp91^phox^ and p22^phox^ protein levels pulled-down with TSPO decreased in the LPS condition relative to vehicle-treated cells, suggesting disruption of these protein-protein interactions under LPS-stimulated conditions. No change was found in the degree of TSPO association with VDAC (*n* = 4–6 independent experiments; TSPO: *p* = 0.036; gp91: *p* = 0.005; p22: *p* < 0.045; VDAC: *p* = 0.743). Data are normalized to vehicle and expressed as mean ± s.e.m. Student’s paired *t* test was performed; *p < 0.05 compared to vehicle-treated microglia. Input and IP images for gp91 are from different blots. Input and IP images for p22 and VDAC are from the same blots, but different exposures. For full blots please see Supplementary Fig. S3

Figure 1a shows western blots of TSPO, gp91^phox^, p22^phox^, and VDAC protein levels in the input fraction (whole cell lysate) under vehicle and LPS-activated conditions. Quantification of proteins in the input fraction by Western blot showed that TSPO, gp91^phox^, and p22^phox^ protein levels significantly increased following LPS activation (100 ng/mL for 18 h) relative to vehicle conditions (Fig. 1b and Supplementary Fig. S3 and S4). VDAC protein levels did not change with LPS-induced activation of microglia (Fig. 1b). To confirm the pattern of increased protein expression in LPS-activated microglia, we performed quantitative real-time PCR under the same conditions. Figure 1c shows that the protein changes following LPS exposure are supported by the gene expression results (Fig. 1c). Notably, LPS concentration (100 ng/ml) and duration of exposure (18 h) used to activate microglia were determined to be non-cytotoxic (Supplementary Fig. S2). Supplementary Fig. S3 shows the full blots shown in Fig. 1. The results of the TSPO co-immunoprecipitation studies using a validated TSPO antibody and detection of TSPO, gp91^phox^, p22^phox^, and VDAC proteins in the TSPO-precipitated fraction is presented in Fig. 1d. Western blot of the TSPO-immunoprecipitated fraction shows that gp91^phox^, p22^phox^, and VDAC are associated with TSPO, providing the first direct evidence of a TSPO association with gp91^phox^ and p22^phox^.

When we compared the level of these proteins in the TSPO-precipitated fraction from LPS- and vehicle-treated microglia, we found that LPS treatment resulted in a significant decrease in the levels of gp91^phox^ and p22^phox^ protein that co-immunoprecipitated with TSPO, with no change in VDAC protein levels (Fig. 1e). Overall, these findings indicate that gp91^phox^ and p22^phox^ are associated with TSPO under normal physiological conditions. However, these interactions appear to be disrupted by microglia activation with LPS since approximately 40% less gp91^phox^ and p22^phox^ protein was immunoprecipitated with TSPO in LPS-treated relative to vehicle-treated microglia. We also confirm that VDAC co-immunoprecipitates with TSPO as previously described [51, 52]. Further, the TSPO association with VDAC was not disrupted by LPS treatment (Fig. 1e). Therefore, based on the TSPO immunoprecipitation results it appears that LPS activation of microglia decreases the association of TSPO with gp91^phox^ and p22^phox^ but not with VDAC, indicating a specific effect of LPS activation on the TSPO-NOX2 subunits interaction.

### Immunofluorescence Confocal Imaging Shows Colocalization of TSPO with gp91^phox^, p22^phox^, and VDAC in Primary Microglia

**Fig. 2:**
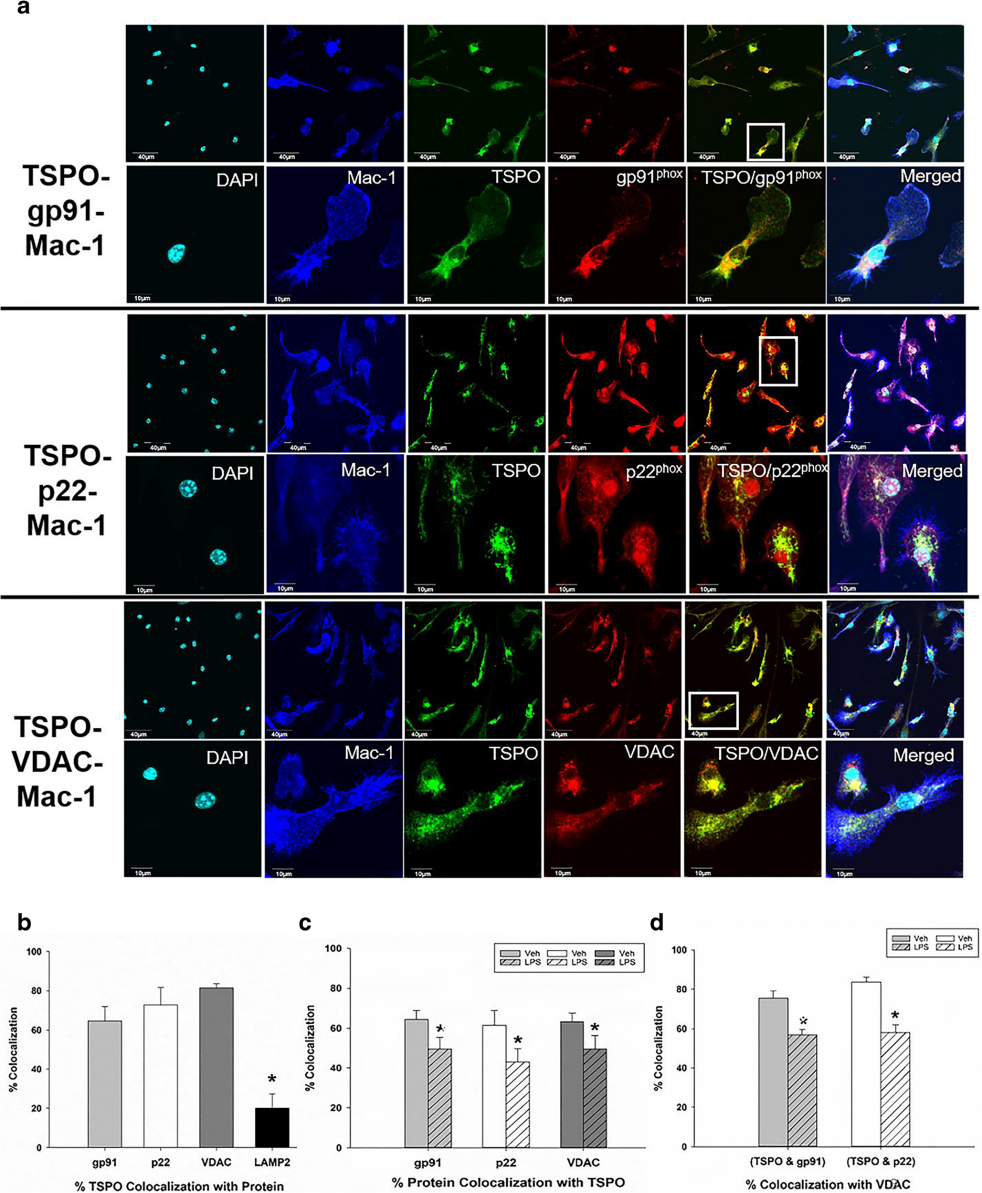
Immunocytochemistry and confocal imaging of TSPO colocalization with gp91^phox^, p22^phox^, VDAC, and LAMP-2 and effect of LPS activation in primary microglia. **a** Triple-label immunofluorescent confocal images of TSPO colocalization with gp91^phox^, p22^phox^, or VDAC in primary microglia (Mac-1 labeled) cells. Confocal images show that TSPO colocalizes with gp91^phox^, p22^phox^, and VDAC in primary microglia. Images in upper panels are at a low magnification: scale bar = 40 μm. Cells in white boxes were selected to be expressed at a higher magnification: scale bar = 10 μm (lower panel). **b** Analyses of protein pair signal colocalization revealed that TSPO has a high degree of colocalization with gp91^phox^ (64.68%), p22^phox^ (72.82%), and VDAC (81.48%) and a lower level of colocalization (20.13%) with the lysosomal marker, LAMP-2 in vehicle-treated microglia. Analysis of variance with Tukey’s post hoc tests shows a significant effect of protein colocalization with TSPO (*F*_3,6_ = 9.8; *p* = 0.0006) where TSPO colocalization with LAMP-2 is significantly lower than with gp91^phox^, p22^phox^, and VDAC (* = *p* < 0.01). **c** The percentage of gp91^phox^, p22^phox^, and VDAC that colocalized with TSPO decreased when microglia were activated with 100 ng/mL of LPS for 18 h relative to vehicle conditions (% gp91 with TSPO: *p* = 0.004; %p22 with TSPO: *p* = 0.002; % VDAC with TSPO: *p* = 0.003). These results indicate that microglia activation disrupts TSPO’s association with gp91^phox^, p22^phox^, and VDAC. **d** Further analysis of signal colocalization indicates that under LPS-stimulated conditions, TSPO associated with gp91 and TSPO associated with p22, exhibit significantly decreased colocalization with VDAC suggesting a movement from the mitochondria to other cellular compartments (% (TSPO with gp91)/ VDAC: *p* < 0.001. % (TSPO with p22) / VDAC: *p* = 0.004). Data are expressed as mean ± s.e.m. *n* = 5–7 independent experiments with > 35 microglia counted per treatment condition per experiment. *n* = 3 independent experiments for TSPO/LAMP-2 labeling with > 35 microglia counted per treatment condition per experiment. Student’s paired *t* test was performed; *p < 0.05 compared to vehicle-treated microglia

To further validate the association of TSPO with gp91^phox^, p22^phox^, and VDAC that we observed with the TSPO co-immunoprecipitation experiments, we performed immunofluorescence confocal imaging of TSPO, gp91^phox^, p22^phox^, and VDAC in vehicle and LPS-treated microglia and measured colocalization of proteins using a computer-based image analysis program (metamorph—see “Methods” section). For these studies, triple labeling of TSPO-gp91^phox^-VDAC or TSPO-p22^phox^-VDAC was performed (see Supplementary Figure S5A-B for representative images of triple labeling). Figure 2a shows TSPO colocalization with gp91^phox^, p22^phox^, and VDAC in primary microglia labeled with Mac-1 at low and high magnifications. The images presented in Fig. 2 were not used for quantification analysis but were used as representative images of the interactions and used Mac-1 to delineate the boundaries of the microglia for presentation purposes. Negative controls in the absence of the primary antibodies for the immunofluorescence confocal studies are provided in Fig. S6A. We also show the absence and presence of TSPO immunostaining in TSPO-KO and wildtype microglia, respectively (Fig. S6B) further confirming the quality of the TSPO antibody. Immunostaining for gp91^phox^ remained present in the TSPO-KO microglia (Fig. S6B).

Figure 2b depicts the quantification of the colocalization of TSPO with gp91^phox^, p22^phox^, and VDAC. We also performed colocalization studies of TSPO with the lysosomal-associated membrane protein-2 (LAMP-2), a lysosomal protein that was not expected to have a high degree of colocalization with TSPO. The results show that the percent colocalization of TSPO with gp91^phox^, p22^phox^, or VDAC was abundant and ranged from approximately 60–80% (Fig. 2b). On the other hand, TSPO colocalization with LAMP-2 was only approximately 20% which is significantly less than the NOX2 subunits and VDAC (Fig. 2b; images in Supplementary Fig. S7). These findings indicate that TSPO has a significantly higher degree of colocalization with proteins that have an outer mitochondrial membrane (i.e., VDAC) or ER (gp91^phox^, p22^phox^) localization than with a lysosomal protein (LAMP-2).

We also compared the colocalization of TSPO with gp91^phox^, p22^phox^, and VDAC in vehicle-treated and LPS-treated primary microglia. We found an effect of microglia activation with LPS on these putative protein interactions. That is, the percent of gp91^phox^ or p22^phox^ that colocalized with TSPO decreased significantly from approximately 60% in vehicle-treated to 40% in LPS-treated microglia (Fig. 2c). A similar effect was observed with VDAC suggesting that the associations of these proteins with TSPO is partly disrupted when microglia are activated with LPS (Fig. 2c). Moreover, when we analyzed the fraction of the TSPO-gp91^phox^ or TSPO-p22^phox^ colocalization that also colocalizes with VDAC, we also observed a decrease with LPS activation (Fig. 2d). The immunofluorescence confocal imaging results are consistent with the co-immunoprecipitation results demonstrating an association of TSPO with gp91^phox^, p22^phox^, and VDAC and this association is disrupted by LPS activation.

### Proximity Ligation Assay Supports a TSPO Interaction with gp91^phox^, p22^phox^, and VDAC in Primary Microglia

**Fig. 3:**
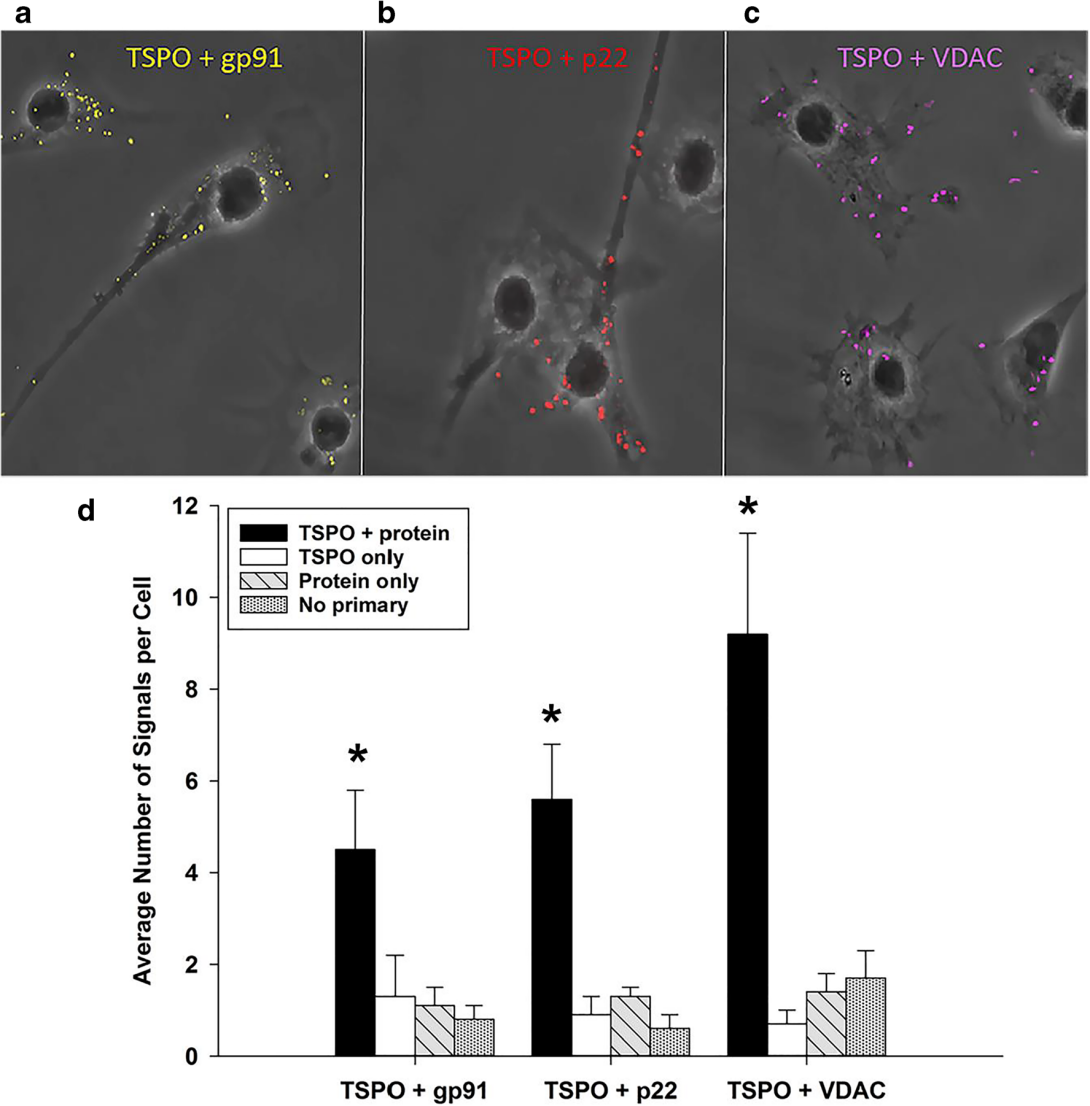
Proximity ligation assay (PLA) supports a TSPO interaction with gp91^phox^, p22^phox^, and VDAC in primary microglia. **a**–**c** Representative PLA phase and pseudo-colored confocal images of protein pair interactions (colored dots) in microglia: TSPO + gp91 (**a**); TSPO + p22 (**b**); TSPO + VDAC (**c**). The images indicate that TSPO interacts with gp91^phox^, p22^phox^, and VDAC respectively supporting the co-immunoprecipitation and immunocytochemistry colocalization results. **d** Quantification of average number of protein pair signals per microglia in vehicle conditions at 18 h. PLA experiments confirms that TSPO interacts with the NOX2 subunits gp91^phox^ and p22^phox^, as well as the mitochondrial protein, VDAC. Imaging and quantification included negative controls for labeling conditions of single antibody only, as well as no primary antibody, to ensure signal specificity. Data are expressed as mean ± s.e.m. *n* = 4–7 independent experiments with > 30 cells counted per treatment and per labeling condition. One-way ANOVA across labeling conditions was performed; (TSPO+gp91: *F*_3,17_ = 8.193, *p* = 0.002; TSPO+p22: *F*_3,11_ = 11.108, *p* = 0.003; TSPO+VDAC: *F*_3,14_ = 9.979, *p* = 0.002). *p < 0.05 = significantly different relative to all other conditions within a protein pair group

To further validate the association of TSPO with gp91^phox^, p22^phox^, and VDAC in primary microglia, we used a proximity ligation assay (PLA) approach. PLA is a powerful method that can detect protein-protein interaction(s) in the native state of cells with high specificity and sensitivity when proteins are in close proximity (30–40 nm apart or less) [53, 54]. Figure 3a–c depicts confocal-phase contrast images of independent TSPO-gp91^phox^, TSPO-p22^phox^, and TSPO-VDAC PLA interactions, respectively, in microglia. The different TSPO-protein interactions are visible throughout the cell as single dots representative of molecular interactions between the proteins providing further support of an association of TSPO with gp91^phox^, p22^phox^, and VDAC. Figure 3d depicts the PLA quantitative results. The results showed specific TSPO associations with the NOX2 subunits gp91^phox^ and p22^phox^, as well as VDAC, confirming the co-immunoprecipitation and immunofluorescence confocal imaging results. Notably, we did not observe an effect of LPS activation in decreasing the number of gp91^phox^ and p22^phox^ interactions with TSPO using the PLA assay (Additional Table S8). The lack of an effect of LPS activation using the PLA assay was due to the fact that the PLA assay is dependent on the primary antibody concentration which was specifically optimized to detect a small fraction of the total protein-protein interactions occurring within a cell.

### The Mitochondria-Associated Endoplasmic Reticulum Membrane (MAM): a Putative Site for a TSPO Association with gp91^phox^-p22^phox^ in Primary Microglia

**Fig. 4:**
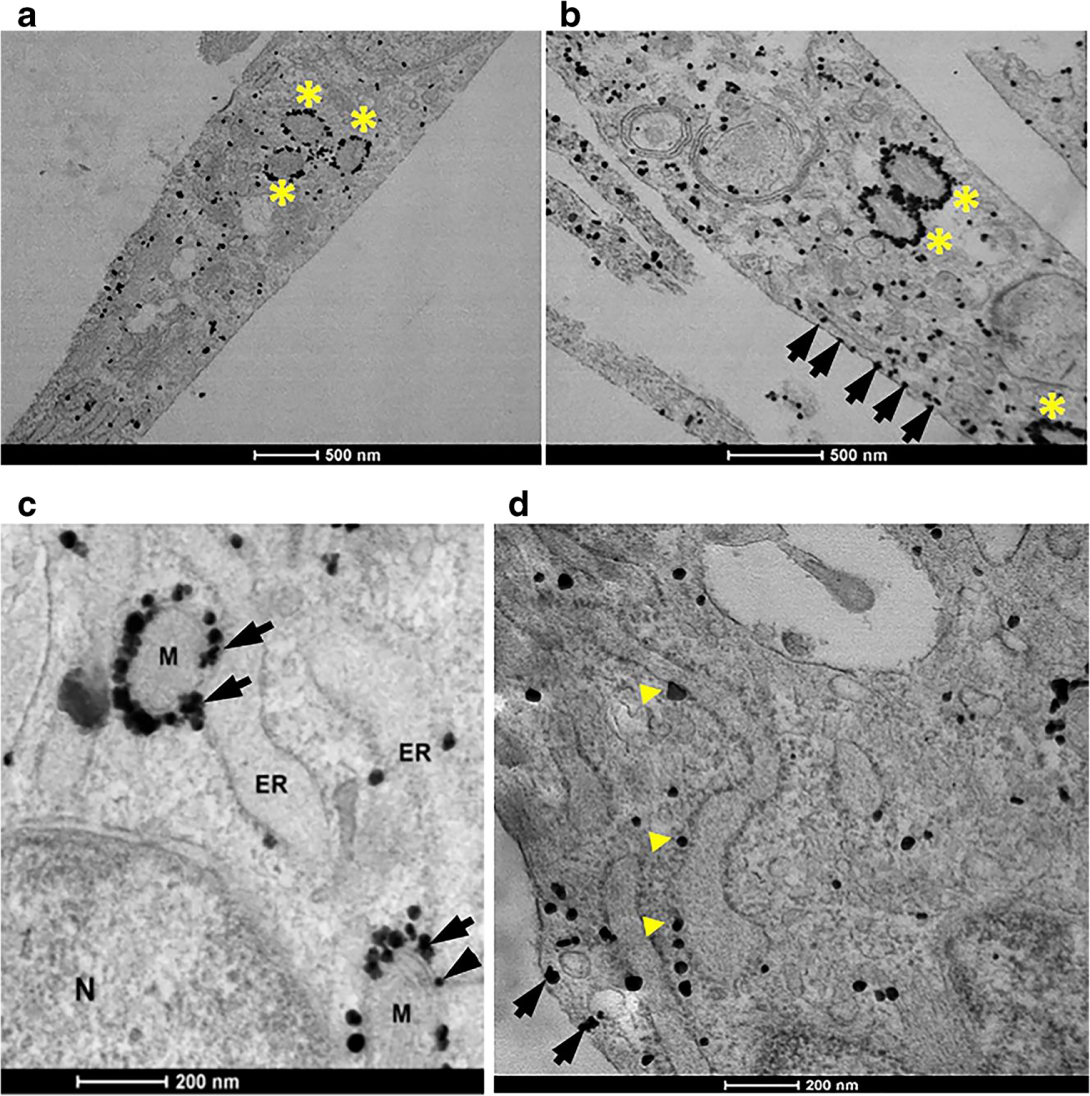
Immuno-gold electron microscopy of TSPO in primary microglia. **a** Immuno-gold electron microscopy of TSPO in vehicle-treated microglia confirms a high level of TSPO expression in mitochondria (yellow stars). Black dots = TSPO. **b** TSPO expression in microglia activated with LPS (100 ng/mL) for 18 h. There is an apparent increase in TSPO expression. TSPO labeling is seen at the mitochondria, as well as other subcellular compartments including at or just below the plasma membrane (black arrows). **c** High magnification imaging of TSPO expression in non-activated microglia indicates TSPO localization in the endoplasmic reticulum (ER) and at the mitochondria-associated ER membrane or MAM (black arrows). N = nucleus; M = mitochondria; ER = endoplasmic reticulum. **d** High magnification imaging of TSPO expression in non-activated microglia shows TSPO localization at the ER (yellow triangles) and at or just below the plasma membrane (black arrows)

It is well established that TSPO and VDAC are proteins that are highly expressed at the outer mitochondrial membrane and that gp91^phox^ and p22^phox^ mature and form the Cytb_558_ heterodimer in the ER. We have previously proposed that these proteins are likely interacting at the mitochondria-associated ER membrane or MAM [55]. To examine whether TSPO is found at the MAM, we performed immuno-gold electron microscopy (IGEM) of TSPO in LPS- and vehicle-treated primary microglia. We used an IGEM approach because the microglia number from culturing is low and not sufficient to obtain enough pure MAM fraction to perform Western blot to confirm purity and assess proteins of interest. Figure 4 depicts images of microglia under normal culture conditions (Fig. 4a, c, d), and after LPS treatment (Fig. 4b). A negative control, that is, the absence of the TSPO primary antibody for the TSPO-IGEM studies is provided in Supplementary Fig. S9. The results presented in Fig. 4a, b demonstrate, as expected, an abundant TSPO labeling of the outer mitochondrial membrane (see yellow stars). With LPS treatment, there is an apparent increase in TSPO labeling in the mitochondria (see yellow stars), as well as TSPO labeling in other subcellular compartments such as the plasma membrane (arrows in Fig. 4b) and throughout the cytoplasm. Figure 4 c and d are higher magnification images of non-activated microglia indicating the presence of TSPO at the MAM (see arrows in Fig. 4c) as well as in the ER (yellow arrow heads in Fig. 4d) and at or just below the plasma membrane (arrows in Fig. 4d). These images confirm that TSPO is highly expressed in the outer mitochondria membrane as previously described. However, our results also provide evidence that TSPO is present in other subcellular compartments such as the plasma membrane and the MAM (see arrows in Fig. 4b–d).

### Modulation of Plasma Membrane TSPO Expression by Reactive Oxygen Species in Primary Microglia

**Fig. 5:**
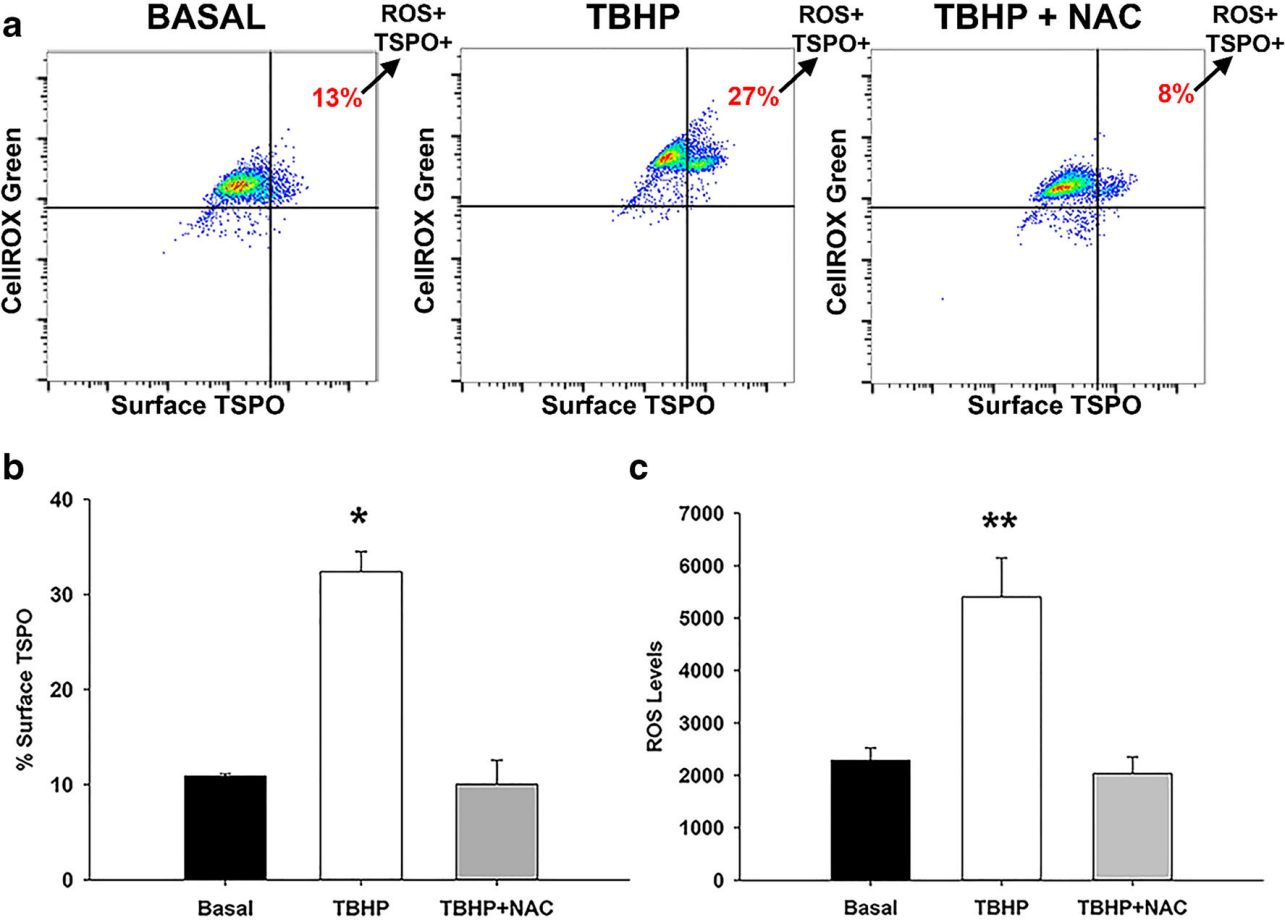
Reactive oxygen species (ROS) modulates surface expression of TSPO in primary microglia cells. **a** Representative flow cytometry dot plots of surface TSPO and CellROX® fluorescence in live primary microglia cells that were treated with and without *N*-acetyl cysteine (NAC), a ROS scavenger followed by tert-butyl hydroperoxide (TBHP), an ROS inducer. Percentage of surface TSPO expression (**b**) and mean fluorescence intensity (MFI) of ROS levels (**c**) with TBHP, ± NAC treatment. Surface TSPO expression and ROS levels increase with TBHP, while NAC treatment inhibited ROS and surface TSPO caused by TBHP. Data are expressed as mean ± sem *n* = 3 independent experiments: One-way ANOVA was performed; *p = 0.0003, *F*_2,6_ = 44.2 compared to basal levels; **p = 0.0046, *F*_2,6_ = 15.1 compared to basal levels

The results of the TSPO subcellular localization studies using TSPO-IGEM in vehicle and LPS-treated microglia suggest that based on the activation state of microglia, there may be an increase in the surface expression of TSPO (Fig. 4). TSPO has been historically known as an outer mitochondrial membrane protein as confirmed by our TSPO-IGEM studies in primary microglia (Fig. 4), although several reports have noted that TSPO is also present at the plasma membrane [56, 57] including our present work using TSPO-IGEM (Fig. 4). Since ROS production is one of the known effects of microglia activation, we hypothesized that an acute burst of ROS may alter the cell surface expression of TSPO. To generate an acute burst of ROS, we exposed primary microglia immediately after harvesting to the ROS inducer tert-butyl hydroxyperoxide (TBHP; 400 μM) for 45 min at 37 °C. The results in Fig. 5a show representative flow cytometry dot plots demonstrating that exposure to TBHP increase ROS levels (Fig. 5b) as well as surface TSPO expression (Fig. 5c) in live microglia selected by SYTOX® staining (see gating scheme in Supplementary Fig. S10). Furthermore, the ROS-induced increase in surface TSPO expression was abrogated by the ROS scavenger *N*-acetyl cysteine (NAC; 5000 μM for 1 h at 37 °C) (Fig. 5b, c).

### TSPO Colocalization with gp91^phox^ in Murine Brain Tissue

**Fig. 6:**
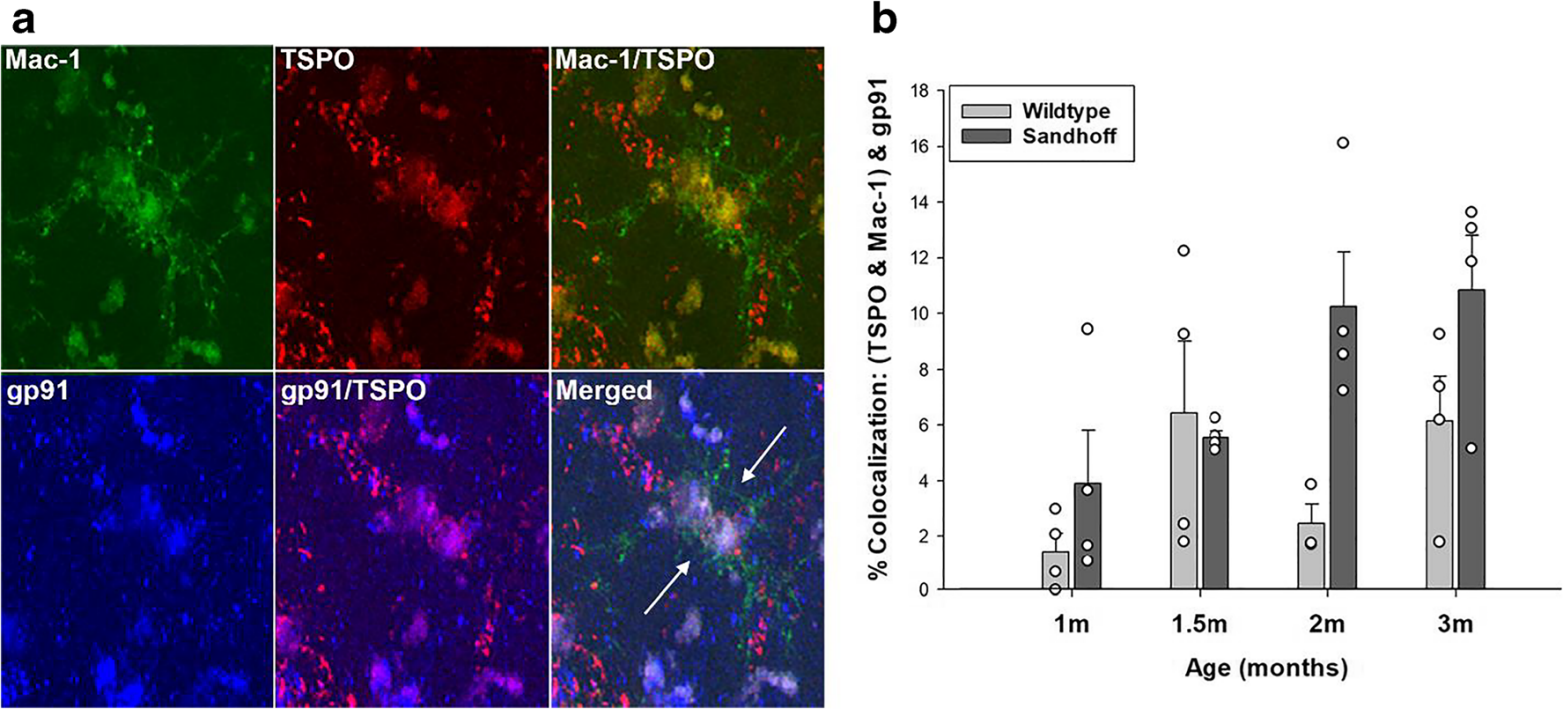
In situ colocalization of TSPO with gp91^phox^ in murine brain microglia. **a** Representative triple labeled immunofluorescent confocal images in the thalamus of a 2-month old Sandhoff disease mouse at 60× with 1.6× zoom. Imaging confirmed that the TSPO signal colocalizes with the gp91^phox^ signal in Mac-1 labeled microglia. **b** Quantification of percent colocalization of TSPO with gp91^phox^ in microglia in the thalamus of Sandhoff disease mice compared to wildtype as a function of age. The data indicates that the TSPO-gp91^phox^ interaction increases as a function of age and progression of neurological disease (age: *F*_3,7_ = 4.257, *p* = 0.017; genotype: *F*_1,7_ = 11.081, *p* = 0.003; age × genotype: *F*_3,7_ = 1.548, *p* = 0.232). Data are expressed as mean ± s.e.m. *n* = 4 independent animals and experiments per group, except for the 2-month old wildtype group which has *n* = 3 due to one animal being excluded as an outlier (> 3 standard deviations from the mean)

The studies presented above were performed in primary microglia. However, to show that the TSPO-NOX2 subunit colocalization also occurs in brain tissue, we performed triple-label immunofluorescence confocal imaging studies of TSPO, gp91^phox^, and Mac-1 in mouse brain tissue. These studies were performed in the brain of Sandhoff disease transgenic and wildtype mice. We used this animal model because we have previously characterized the TSPO response in microglia and astrocytes that occurs as a result of progressive neurodegeneration in this murine model of Sandhoff disease [47]. Panel a in Fig. 6 shows that TSPO and gp91^phox^ colocalize in microglia in the mouse brain. Thus, the TSPO-gp91^phox^ colocalization not only occurs in primary microglia in culture but also in situ in the brain. Furthermore, the TSPO-gp91^phox^ colocalization in the thalamus of Sandhoff disease mice increases as a function of age and progression of neurodegeneration in this preclinical animal model of neurodegeneration (Fig. 6b). It should be noted that given this is a model of neurodegeneration, and that there has been documentation of a compromised blood brain barrier in this murine model of Sandhoff’s disease [58]. It is possible that some cells labeled with Mac-1 could also represent infiltrating peripheral immune cells that would also stain positive for Mac-1.

### Bioinformatics Reveal Structural Elements for Heme or Porphyrin Transit from the Intermembrane Space to the Cytosolic Surface of the Outer Mitochondrial Membrane

**Fig. 7:**
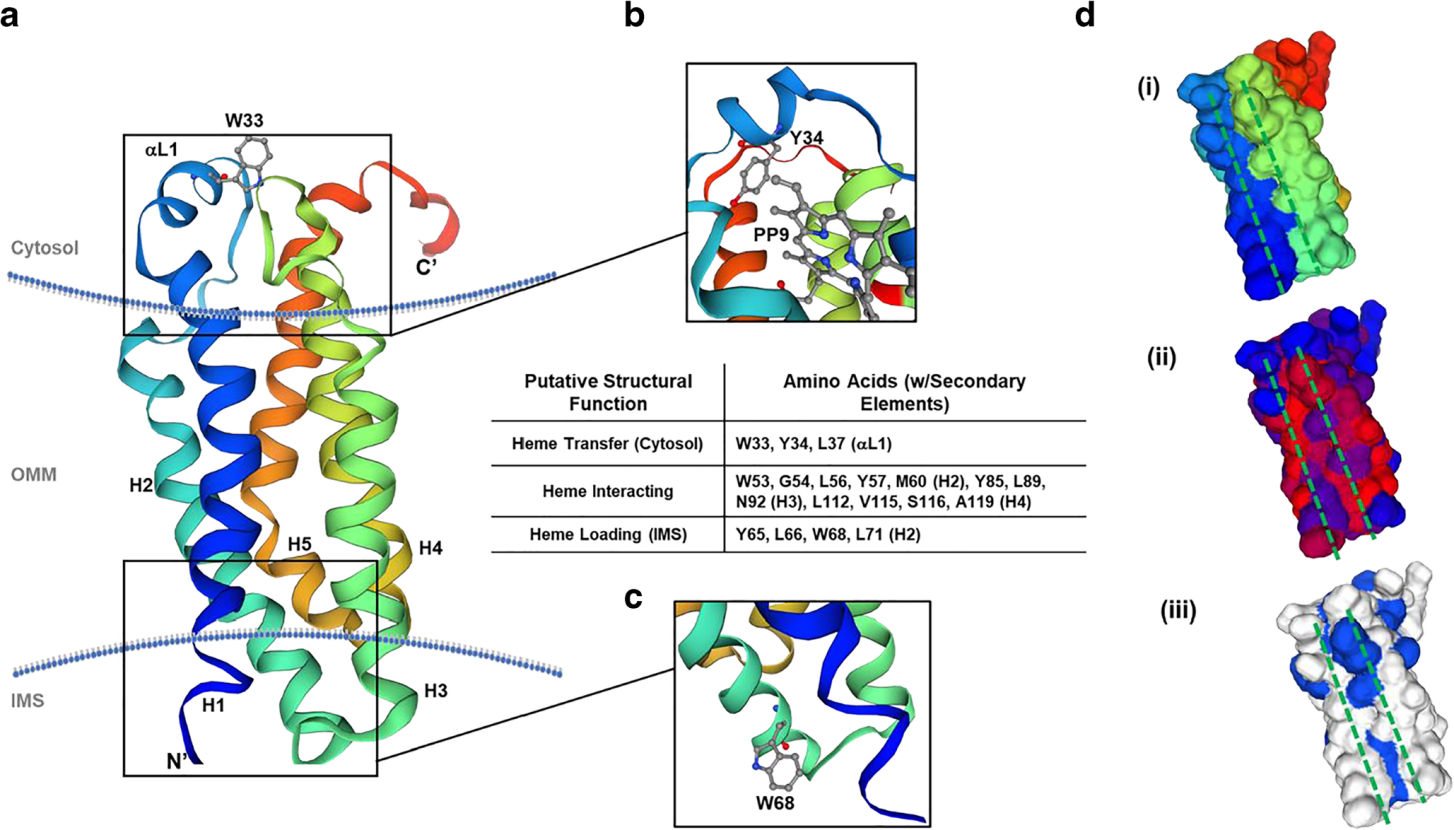
Bioinformatics-based modeling of the hTSPO monomer and putative heme-binding capabilities. (**a**) A ribbon diagram of hTSPO mapped to mouse TSPO structure (PDB: 2mgy.1) with a-helices color coded (blue: Helix 1, blue-green: Helix 2, green: Helix 3, green-yellow: Helix 4, orange-red: Helix 5). The N-terminus (N′) is labeled along with the C-terminus (C′) and conserved tryptophan-33 (W33) in the cytosolic a-loop. (**b**) immediately below W33 is the proposed porphyrin-binding site identified in crystal structures of *Rhodobacter sphaeroides* TSPO illustrated by the appearance of protoporphyrin IX (PP9). The conserved a-loop motif of WYXXLXKP, wherein W33, Y34, and L37 of hTSPO were identified as potential heme-interacting residues. Using the HemeBind software and the sequence and modeled structure of hTSPO, we were able to identify amino acids with the potential for heme binding that were not implicated in porphyrin-binding in previous models. Including a motif (WXXLYXXM) in the intermembrane space of the mitochondria that could be the site of heme loading at the close of biosynthesis typified by the presence of W68 in hTSPO at the bottom of helix 2 (**c**). (**d**) An examination of the predicted protein surface topology using the same colors as the ribbon structure reveal a groove or trough between helices 1 and 3 (i). This trough has stretches of hydrophobicity (ii, red) and aromaticity (iii, blue) that resemble traits of other heme binding proteins without conserved or strong heme-binding motifs

The relevance of the proposed TSPO-NOX2 interaction may be founded in a fundamental element of NOX2 function, heme. Heme biosynthesis is finalized within mitochondria [59], and NOX2 subunits have shown localization on ER-derived membranes including phagosomes, specialized endosomes (“redoxisomes”), and at the leading edge of lamellipodia [60]. Because the contiguous nature of ER-mitochondrial physiology [61], we employed bioinformatic structural analyses to determine if TSPO may provide a conduit for heme to reach gp65, the heme-accepting immature precursor of gp91^phox^. Using SWISS-MODEL workspace [48], we modeled human TSPO (hTSPO) sequence (Uniprot Identifier: P30536-1) onto a known crystal structure for mouse TSPO (mTSPO) with a diagnostic ligand, PK11195, bound to a putative porphyrin-binding site (PDB: 2mgy.1) [62, 63]. The structural modeling produced a transmembrane protein structure with five helices, a loop on the cytosolic surface of the outer mitochondrial membrane (OMM), a short N-terminal region residing in the intermembrane space (IMS), and a longer C-terminal region extending into the cytosol (Fig. 7a). This structure was similar to previously reported monomeric structures of TSPO in other species [62–65]. An analysis of the primary sequence and putative structure of hTSPO were examined for the presence of hallmark heme-binding motifs, and none were found; however, not all heme-interacting proteins contain these elements [66]. Thus, the structure and sequence were used with the HemeBind software [50] to ascertain if residues capable of binding heme were present within the protein. According to the HemeBind analysis, 18 amino acids were identified that could interact with heme (Fig. 7, table inset), and these amino acids were restricted to helices 2, 3, and 4, but three additional amino acids (W33, Y34, and L37) resided on the cytosolic loop (Fig. 7b). The triplet was part of a larger conserved WYXXLXKP motif among hTSPO, mTSPO, and TSPO from *Rhodobacter sphaeroides* (rTSPO) that was crystalized with protoporphyrin IX (PPIX) (Supplemental Fig. S11) [49]. Mapping the PPIX binding site of rTSPO onto hTSPO indicates that Y34 of the loop WYXXLXKP motif extends towards PPIX, while W33 faces the cytosol (Fig. 7b). While the porphyrin-binding site and a cytosolic facing element could be present, there was little evidence to support how a molecule might reach portions of TSPO near the OMM. Continued analysis of putative heme-interacting residues unearthed a motif in the IMS of hTSPO with surface residues that may bind heme; these include Y65, L66, W68, and L71, which are part of a conserved WXXLYXXM motif at the bottom of helix 2 near an IMS loop. In fact, modeling predicts that W68 can extend into the IMS away from helix 2 towards making it available to proteins or molecules in the IMS (Fig. 7c). To discern if the modeled structure could provide insight into any connectivity between the proposed IMS WXXLYXXM heme-loading motif, the porphyrin-binding site, and the OMM WYXXLXKP site, we modeled the surface topology of hTSPO to determine if structural elements were present to connect these elements (Fig. 7d (i)). We used predicted protein hydrophobicity (Fig. 7d (ii)), as an indication of nonpolar amino acids, and aromaticity (Fig. 7d (iii)), to demonstrate surface aromatic amino acids on hTSPO. Both nonpolar and aromatic amino acids have been shown to be critical to heme-interactions in heme-proteins lacking strong, hallmark heme-binding motifs [66]. Our examination found that there were aspects of protein hydrophobicity, from the presence of nonpolar amino acid involved in potential heme-interactions stretched from the IMS to the porphyrin-binding pocket (Fig. 7d (ii), red). This trough included predicted heme-interacting residues G54, L56, L89, L112, V115, and A119. The area also surrounded polar amino acids M60, N92, and S116 also implicated in heme-binding by our bioinformatics approach. Next, the aromaticity revealed a significant aromaticity near the porphyrin-binding pocket (Fig. 7d (iii), blue), but there were also increased levels of aromaticity surrounding the proposed heme-loading WXXLYXXM motif and the cytosolic loop WYXXLXKP motif above the porphyrin-binding site (Fig. 7d (iii), blue). Taken together, these results demonstrate a strong possibility for hTSPO to interact with heme and/or other porphyrins, and there may be physicochemical properties that may allow heme to move from the IMS to the cytosol along the lateral surface of the protein monomer.

## Discussion

There are several novel findings resulting from the present study. First, using three different experimental techniques, we demonstrate a novel interaction of TSPO with the NOX2 subunits gp91^phox^ and p22^phox^ in primary microglia. We also confirmed that TSPO associates with VDAC as previously described [51, 52]. Based on our TSPO-IGEM results, we confirm that TSPO is present at the outer mitochondria membrane, but also at other subcellular compartments such as the MAM and the plasma membrane. We have hypothesized that the MAM may be a subcellular site where TSPO may associate with the NOX2 subunits (Fig. 4) since the MAM is a site of ER-mitochondria communication [61].

Second, we show that the TSPO association with gp91^phox^ and p22^phox^ appears to be disrupted by LPS treatment of microglia. This is demonstrated by the fact that the relative amount of gp91^phox^ and p22^phox^ protein that co-immunoprecipitates with TSPO in LPS-treated microglia decreases relative to vehicle-treated microglia despite the fact that the LPS treatment of microglia increases TSPO, gp91^phox^, and p22^phox^ protein levels in the input fraction (Figs. 1 and 2). It is possible that the TSPO association with gp91^phox^ and p22^phox^ is transient and depends on the state of microglia activation since the TSPO interaction with VDAC did not change with LPS treatment (Fig. 1). Parenthetically, the latter is consistent with other studies in which cellular treatments that increase TSPO expression do not change VDAC levels [67] and VDAC expression does not change in TSPO-KO mice [68].

Third, we provide evidence that exposing microglia to an acute burst of ROS increases the cell surface expression of TSPO, an effect that is abrogated by an ROS scavenger (Fig. 4 and Fig. 5). Consistent with our current findings that TSPO is present in the cell surface of primary microglia, a recent study using the BV2 microglia cell line has shown that TSPO is present in the plasma membrane using differential centrifugation and analysis of subcellular fractions by Western blot [69]. This study also showed that LPS stimulation of BV2 cells increased TSPO levels in mitochondria by approximately 30% but increased plasma membrane TSPO by 100% with minimal levels of TSPO present in cytosolic or nuclear fraction with or without LPS. The functional significance of an ROS- or LPS-induced increase in TSPO cell surface expression is not known, but we hypothesize that it may be due to the association of TSPO with the NOX2 subunits. What is not known, is the subcellular source of the increase in cell surface TSPO observed following a burst of ROS and following LPS treatment. Future studies will specifically address this important question. Finally, we provide evidence that the TSPO-gp91^phox^ subunit colocalization is also observed in brain tissue microglia from a murine model of neurodegeneration suggesting that these associations not only occur in primary microglia but also in the brain in situ (Fig. 6).

While the current study provides multiple lines of evidence supporting an association of TSPO with gp91^phox^, p22^phox^, and VDAC, it has limitations. First, most of the studies presented here are in vitro studies and there is no in vitro culturing method that can recapitulate all of the hallmark features of neonatal or adult microglia in brain tissue [70]. Additionally, while in vitro studies are useful for defining mechanism(s) and determining cellular specificity of protein associations, as we have done in the present study, they also have limitations given that microglia are operating without feedback from other brain cells such as neurons and astrocytes. The present work in primary microglia from neonatal mouse brain is an important starting point in order to understand the function of TSPO which is currently unknown in microglia. Using primary microglia is an appropriate approach and a significant improvement over using cell lines which can respond differently to certain modes of activation relative to primary microglia [71, 72]. Finally, while there is evidence that neonatal microglia may differ in behavior from adult microglia [73], microglia from the adult brain are more limited in number than microglia from the neonatal brain. Thus, despite the limitations outline, using neonatally derived microglia, is a suitable starting point to begin to understand the function of TSPO.

Evidence of a TSPO-NOX interaction has also recently been described by Gatliff et al., [51]. The study by these investigators indicates that TSPO modulation of mitochondrial calcium signaling via VDAC activates another NOX subtype, the calcium-dependent NOX5. However, NOX5 is not expressed in phagocytic cells including microglia [32, 74, 75]. Thus, the TSPO-mediated calcium-dependent activation of NOX5 is different from our present study in which we demonstrate an association between TSPO and the NOX2 subunits gp91^phox^ and p22^phox^. Another distinctive difference between the work of Gatliff et al. [51] and ours is that they used cell systems such as mouse embryonic fibroblasts, canine mammary gland epithelial cells, and the neuronal cell line SH-SY5Y compared to primary microglia in our study. The cell types used in the Gatliff et al.’s study were in some cases transformed or neuronal cell lines with functions that are different to microglia. It is well established that glial cells (microglia and astrocytes) are the principal contributors of the increase in TSPO levels in the brain neuropil following diverse brain pathologies [3]. Therefore, the TSPO-NOX2 interaction that we have identified in primary microglia is vastly different from the TSPO-mediated, calcium-dependent modulation of NOX5 activity [51].

During the review of our current manuscript, another study was published describing a TSPO-NOX1 interaction [76]. In this study, they find that laser photocoagulation injury of the retina results in activation of resident microglia and increased microglia TSPO levels. The increase in TSPO levels results in a TSPO-mediated increase in cytosolic calcium concentrations essential for the activation of NOX1 with a subsequent increase in extracellular ROS production. The increase in extracellular ROS results in the loss of photoreceptor cells and retinal injury. These two previous studies, along with our current work, indicate a relationship between TSPO and different NOX enzyme subtypes in microglia implicating TSPO in the regulation of NOX activity and ROS production in microglia.

Our current findings raise several important questions on the association of TSPO with gp91^phox^ and p22^phox^ in primary microglia. One relevant question is the molecular basis for an association between TSPO and NOX2. We previously hypothesized [55] that an association between TSPO and NOX2 subunits may be related to the ability of TSPO to bind both heme [77, 78] and cholesterol [21, 79] and the absolute requirement for heme to dimerize gp91^phox^ and p22^phox^ to form a functional Cytb_558_ heterodimer. That is, the NOX2 subunits gp91^phox^ and p22^phox^ that we demonstrate to associate with TSPO are known to dimerize via two heme molecules to form the membrane-bound Cytb_558_ [33–36]. In the absence of heme, these subunits dissociate and are degraded by the proteasome pathway [34]. Cholesterol is also needed for the NOX2 cytosolic subunits to translocate to the plasma membrane and form an active NOX2 complex [38, 39].

**Fig. 8:**
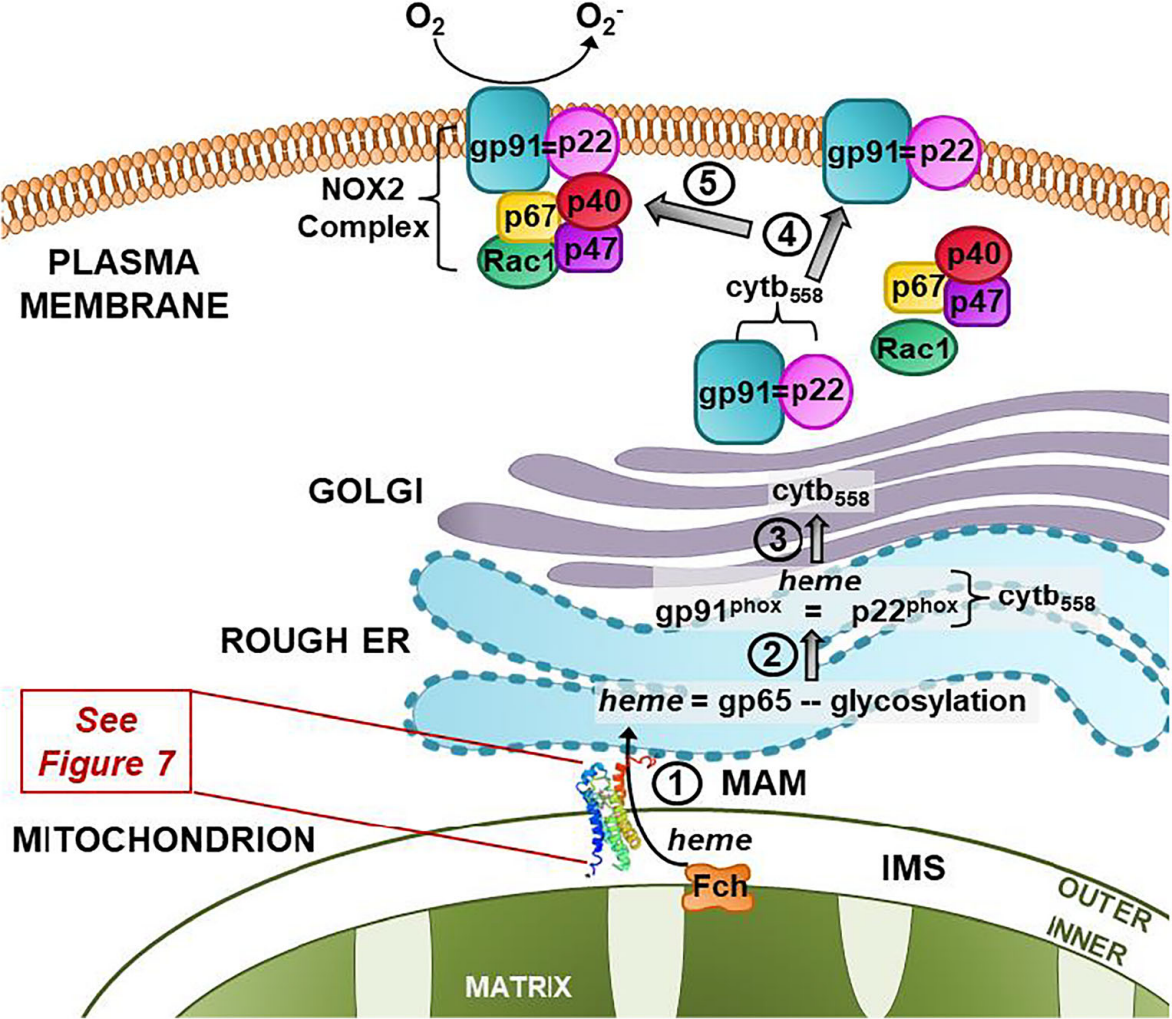
Working model on the functional significance of a TSPO-NOX2 association in microglia: In this model, we propose that TSPO in the outer mitochondria membrane is able to (1) transfer ferrochelatase synthesized heme from the inner mitochondri membrane to the gp65 precursor of gp91^phox^. Once heme has been delivered to gp91^phox^, then (2) it is able to dimerize with p22^phox^ in the ER to: (3) form the gp91^phox^ = p22^phox^ and principal catalytic subunit of NOX2, cytochrome b_558_ (Cytb_558_). Once Cytb_558_ forms, then (4) it is able to traffic to the plasma membrane/lipid rafts where it remains dormant until an activating event takes place. (5) NOX2 activation promotes the translocation of the cytosolic subunits p40, p47, p67, and Rac1 to the plasma membrane to form an active NOX2 complex. MAM: mitochondrial associated membrane; Fch: ferrochelatase; IMS: intermembrane space

Another question raised by our present study relates to the subcellular site(s) and the functional role(s) of a TSPO-NOX2 interaction. Studies have shown that the MAM is a preferential subcellular site for heme transport to heme acceptor proteins such as cytochrome P450 [80]. TSPO is a protein that is present at the MAM (Fig. 4) and binds heme [77, 78]. Thus, it is biologically plausible, and we propose that in microglia (Fig. 8), TSPO may be able to transfer mitochondria-synthesized heme from ferrochelatase (FECH), the enzyme that synthesizes heme in the inner mitochondria membrane to heme-accepting proteins in the ER such as gp65, the precursor of gp91^phox^ (Figs. 7 and 8). TSPO is an outer mitochondria membrane protein that is strategically localized in close proximity to FECH in the inner mitochondrial membrane. Based on their subcellular localizations, it is biologically plausible that FECH can directly transfer mitochondria-synthesized heme to TSPO for transport out of mitochondria to heme-accepting proteins such as gp65, the precursor of gp91^phox^ in the ER via the MAM (Figs. 7 and 8).

What is the evidence that TSPO is a heme-binding protein and that it can translocate heme and porphyrins across the mitochondria membrane? The original description that porphyrins and metalloporphyrins are endogenous TSPO ligands was made over three decades ago [78]. However, there is a paucity of knowledge on the physiological function of TSPO binding of heme and porphyrin. A study using radiolabeled [^55^Fe]-heme showed that heme binds to TSPO with high affinity [77]. That is, Cos-1 cells transfected with TSPO cDNA increased levels of [^3^H]-PK11195 binding and [^55^Fe]-heme-binding as compared to those transfected with antisense. The binding of [^55^Fe]-heme to TSPO in transfected Cos-1 cells and isolated mouse liver mitochondria had a Kd of 12 nM and was displaceable by the TSPO ligands PK11195, Ro5-4864, and protoporphyrin IX. The authors summarize that TSPO plays a role in controlling the trafficking of porphyrins and heme by the uptake of the porphyrins and the transport of heme out of the mitochondria. Further support for TSPO having a function in heme transport comes from studies in primitive erythropoiesis [81]. In chickens, a TSPO homolog *PBRL* is solely expressed during the early development of differentiating erythrocytes and the expression is highly correlated with hemoglobin genes. This study showed that the treatment of chicken embryos with the prototypical TSPO ligand PK11195 resulted in decreased levels of all main embryonic globins without affecting housekeeping genes. The authors indicate that *PBRL* plays a role in globin protein levels by regulating heme levels during primitive erythropoiesis. They note that collectively their findings support a putative role of *PBRL* in regulating heme availability for hemoglobin assembly [81].

Another study examining the effect of global TSPO knockout in mice showed that in bone marrow, from all of the heme-synthesizing enzyme measured, there was a selective increase in the gene expression of FECH [68]. The increase in FECH in bone marrow from TSPO-KO mice was associated with a significant increase in bone marrow heme concentration. This study suggests a potential relationship between TSPO and FECH.

Although the physiological basis for a NOX2-TSPO interaction may involve the transfer of heme to NOX2, one obstacle to this hypothesis is the absence of a transit mechanism for heme out of mitochondria to NOX2 on ER-endosomal membranes. While TSPO does possess a porphyrin-binding site, TSPO structures do not appear to have transporter or channel elements. Our model of hTSPO identifies 18 amino acids that may be involved in heme-interactions, but none are part of established heme-binding motifs. This is not unusual for heme-binding proteins [66], as the human intestinal heme transporter lacks the hallmark motifs associated with heme-binding [82]. The organization of three tryptophan residues (W33, W53, and W68) in our hTSPO model reveals a pathway of hydrophobicity and aromaticity on the lateral surface of hTSPO that could participate in heme transit. Tryptophan residues are crucial for heme-interactions and shaping heme protein conformations [83]. In our model, W68 is located at the bottom of helix 2 in the IMS proximal to the last enzyme in heme biosynthesis, FECH [84]. Here, it is anticipated that FECH releases heme into the IMS where it interacts with the W68-containing motif (WXXLYXXM). The heme migrates along a “trough” created by nonpolar and aromatic amino acids, including W53, between hTSPO helices [50, 66] en route to the porphyrin-binding pocket (Fig. 7d (ii)). Near the porphyrin-binding pocket, the hydrophobicity narrows, and the aromaticity increases moving towards the cytosolic surface of hTSPO (Fig. 7d (iii)). Finally, a motif (WYXXLXKP) containing W33 in the cytosolic a-loop could be the site of heme presentation to gp91^phox^ (Fig. 7a). This would likely involve the transfer of heme from the porphyrin-binding pocket to nonpolar L37 and aromatic Y34 and onto W33 (Fig. 7b). We found no histidine residues along the path that might slow or impede heme transit by binding to the molecule [85, 86]. Consequently, the route identified on the lateral surface of hTSPO would allow heme to move from the IMS to the OMM surface near gp91^phox^.

Collectively, our present findings indicate that TSPO associates with and may be able to regulate the expression of one of the principal NOX2 subunits (i.e., gp91^phox^) and thus, be able to modulate Cytb_558_ formation, NOX2 activity, and redox homeostasis in microglia. This working model is consistent with a known function of TSPO in the plant *Arabidopsis thaliana* in which abiotic stress regulates the homologous *TspO* [87]. Abiotic stress in *A. thaliana* increases TSPO levels. Further, in *A. thaliana*, TSPO regulates the cell surface expression of the aquaporin PIP2;7 by interacting with PIP2;7 at the ER and Golgi membranes [88]. The function of TSPO in *A. thaliana* provides a mechanism by which TSPO can modulate PIP2;7 expression at the plasma membrane.

## Conclusions

We provide new evidence of a novel association of TSPO with gp91^phox^ and p22^phox^ in primary microglia. We hypothesize that this complex may be present at the MAM to mediate the transfer of mitochondrial heme to the gp65 precursor of gp91^phox^. This molecular mechanism would confer a modulatory role for TSPO in the maturation of gp91^phox^. In this way, TSPO may be able to regulate the heme-containing and principal catalytic subunit of NOX2, its enzymatic activity, ROS production, and microglia redox homeostasis.

## Electronic Supplementary Material

ESM 1 (DOCX 12044 kb)

## Abbreviations

TSPO: translocator protein 18 kDa
hTSPO: human translocator protein 18 kDa
rTSPO: rat translocator protein 18 kDa
mTSPO: mouse translocator protein 18 kDa
NOX2: NADPH oxidase 2
LPS: lipopolysaccharide
VDAC: voltage-dependent anion channel
ER: endoplasmic reticulum
ROS: reactive oxygen species
KO: knockout
mPTP: mitochondrial permeability transition pore
Cytb_558_: flavocytochrome b_558_
TBHP: tert-butyl hydroperoxide
NAC: *N*-acetyl cysteine
Mac1: macrophage integrin 1
PP9 or PPIX: protoporphyrin IX
MAM: mitochondrial associated membrane
ALS: amyotrophic lateral sclerosis
PN: post-natal day
FBS: fetal bovine serum
IMPC: International Mouse Phenotyping Consortium
EMMA: INFRAFRONTIER/European Mouse Mutant Archive
IP: immunoprecipitation
PVDF: polyvinylidene difluoride
TBS: Tris-buffered saline
ROIs: regions of interest
PLA: proximity ligation assay
RCA: rolling circle amplification
PFA: paraformaldehyde
PBS: phosphate-buffered saline
MFI: mean fluoresence intensity
SEM: standard error of the mean
LAMP-2: lysosomal-associated membrane protein-2
IGEM: immuno-gold electron microscopy
OMM: outer mitochondrial membrane
IMS: intermembrane space
