# Supplementary material for: A Novel Interaction of Translocator Protein 18 kDa (TSPO) with NADPH Oxidase in Microglia
Source: Mol Neurobiol. 2020 Aug 2;57(11):4467–87. doi: 10.1007/s12035-020-02042-w (PMC7515859; doi:10.1007/s12035-020-02042-w)
Supplement: Supplementary file 1 — (DOCX 12044 kb) [file 12035_2020_2042_MOESM1_ESM.docx]

**A Novel Interaction of Translocator Protein 18 kDa (TSPO) with NADPH Oxidase in Microglia**

Meredith K. Loth^1^, Sara R. Guariglia^1^, Diane B. Re^1^, Juan Perez^2^, Vanessa Nunes de Paiva^2^, Jennifer L. Dziedzic^2^, Jeremy W. Chambers^2^, Diana J. Azzam^2^, Tomás R. Guilarte^1,2*^

^1^Department of Environmental Health Sciences

Mailman School of Public Health

Columbia University, New York, NY. USA

^2^Department of Environmental Health Sciences

Robert Stempel College of Public Health & Social Work

Florida International University, Miami, Florida. USA

**
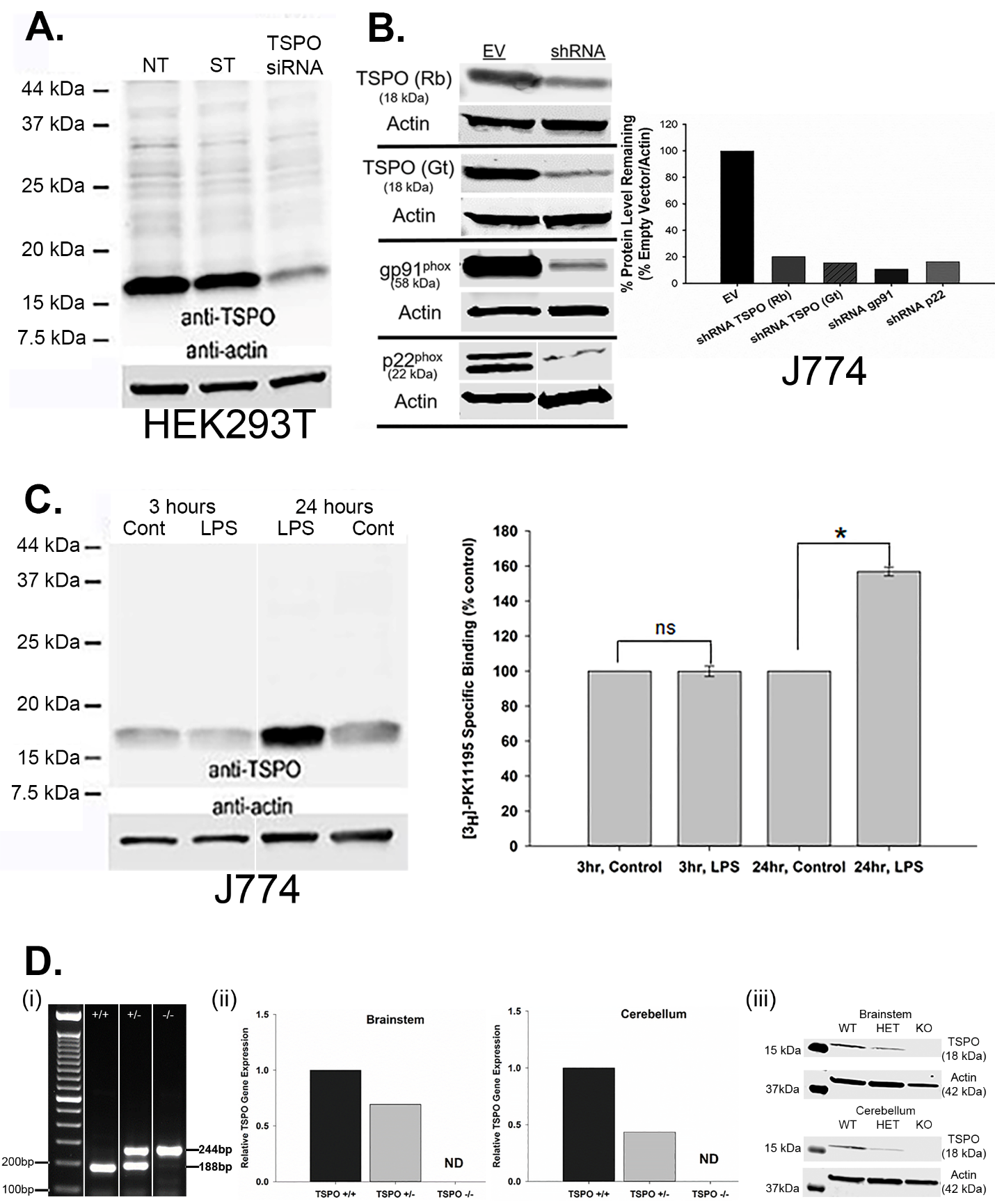
**

**Supplemental Figure S1:** Antibody Validation (A) TSPO Western blot of human embryonic kidney cells (HEK293T) not transfected (NT), transfected with a scramble siRNA (ST), or with a TSPO siRNA. There was a significant 70% decrease of TSPO protein (MW: 18kDa) by the TSPO siRNA. (B) Gene silencing by transduction of shRNA lentiviral particles in a J774 murine macrophage cell line. The figure to the right shows the degree of protein level reduction relative to empty vector (EV) for TSPO (2 different antibodies; Rb=rabbit; Gt=goat), gp91^phox^, and p22^phox^ (from non-adjacent lanes on same gel). There was an 80-90% decrease in protein levels relative to EV. (C) Effect of LPS treatment of J774 cells on TSPO protein expression. TSPO protein levels in J774 cells that were exposed to LPS (1 μg LPS/ml media) for 3 or 24 hrs. Both time points were run on the same gel, however, the 3 hr lanes were non-adjacent to the 24 hr lanes. There was a significant 60% increase of TSPO protein by LPS at 24 hrs with no change at 3 hrs. The graph on the right shows that the TSPO protein data was confirmed using [^3^H]-R-PK11195 receptor binding (60% increase in specific binding) at 24 hr with no increase at 3 hr (n=3). (D) TSPO antibodies were further validated using wildtype (WT; +/+), heterozygous (HET; +/-), and global TSPO knockout (KO; -/-) mice. We show the genotyping of the mice from non-adjacent lanes on same gel (i), mRNA levels by qRT-PCR (ii), and protein data by Western blot (iii) in two different brain regions (brainstem and cerebellum), demonstrating a TSPO gene dosage effect with no detection of TSPO gene or protein expression in the TSPO-KO brain tissue. Data are normalized to vehicle, control, or wild type condition and expressed as mean ± s.e.m. Student’s t-test: *p<0.05 compared to control


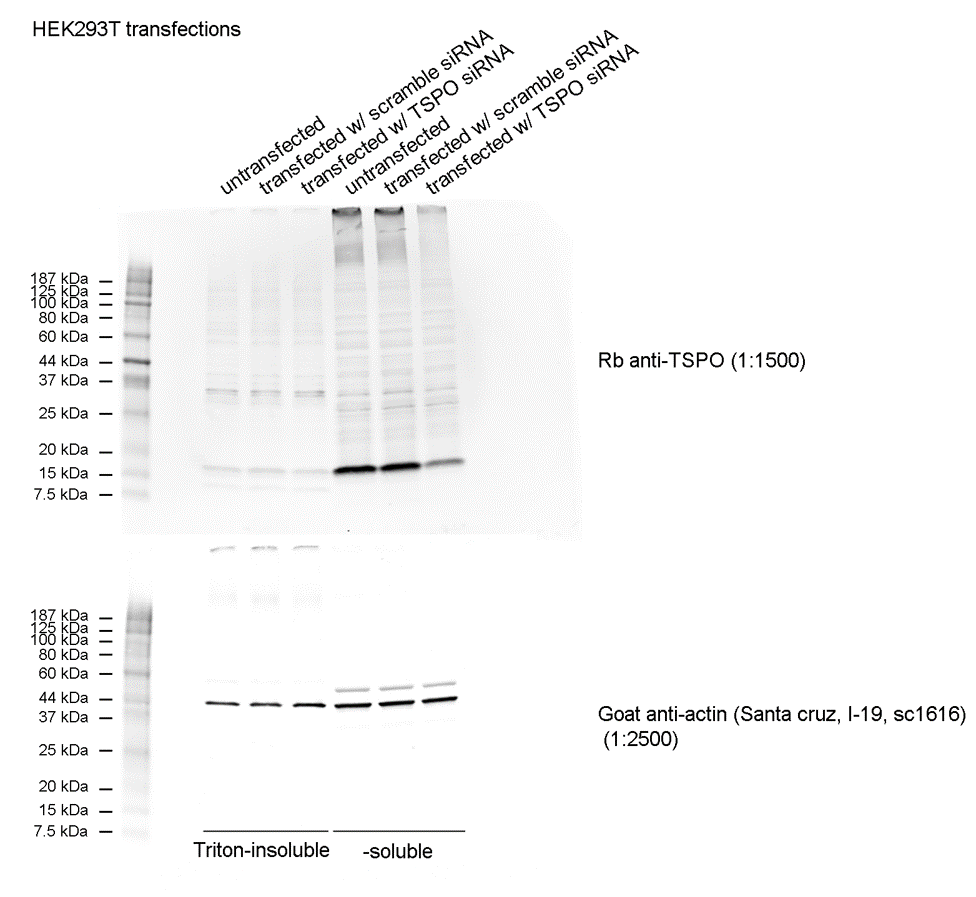
**Full Blot from SF 1A**

**
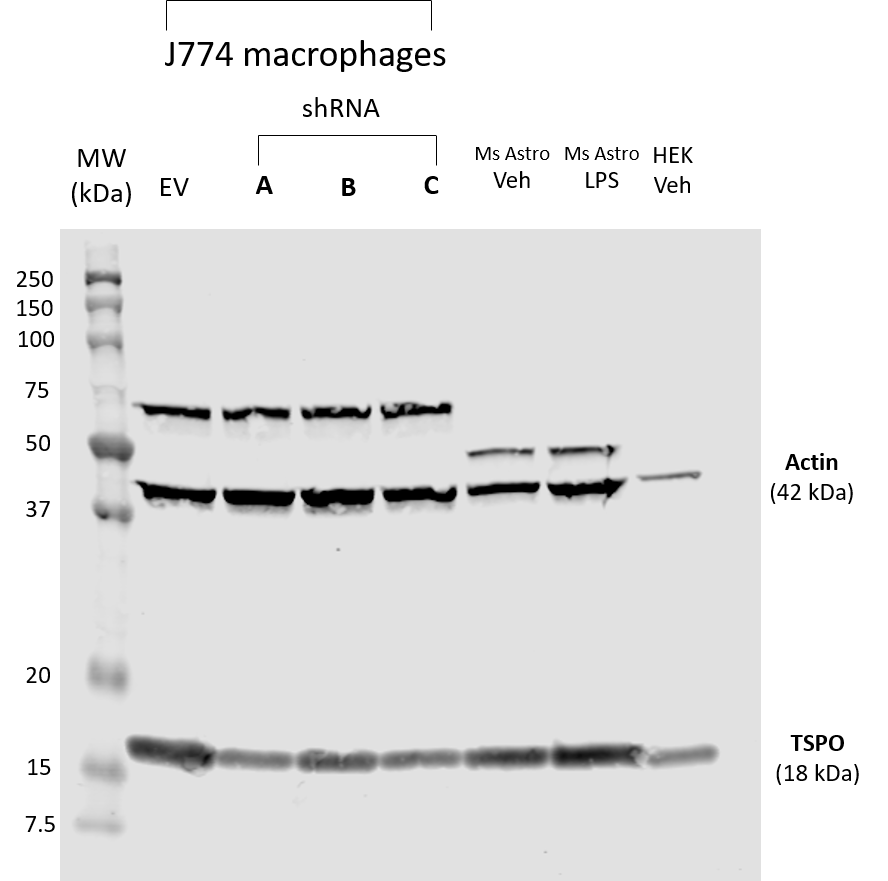
**
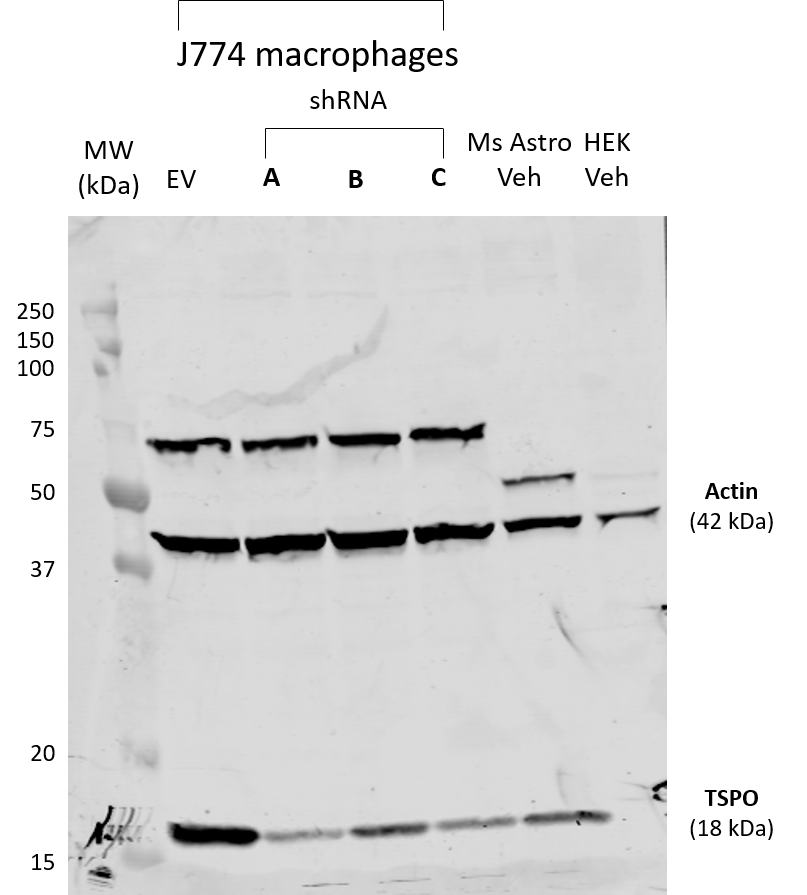
**Full Blot from SF 1B (TSPO, Rb) Full Blot from SF 1B (TSPO, Gt)**

**
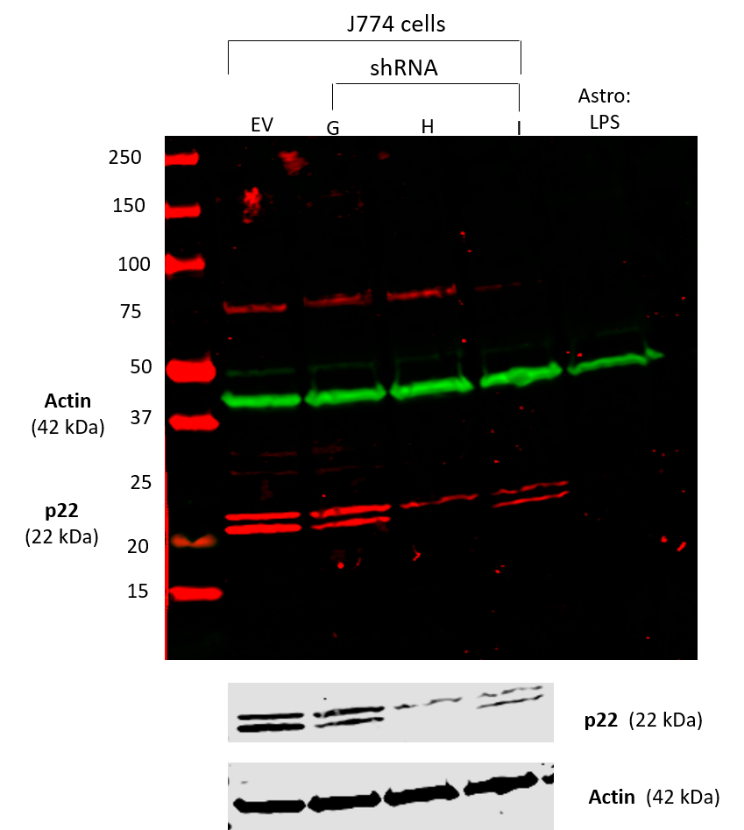
**
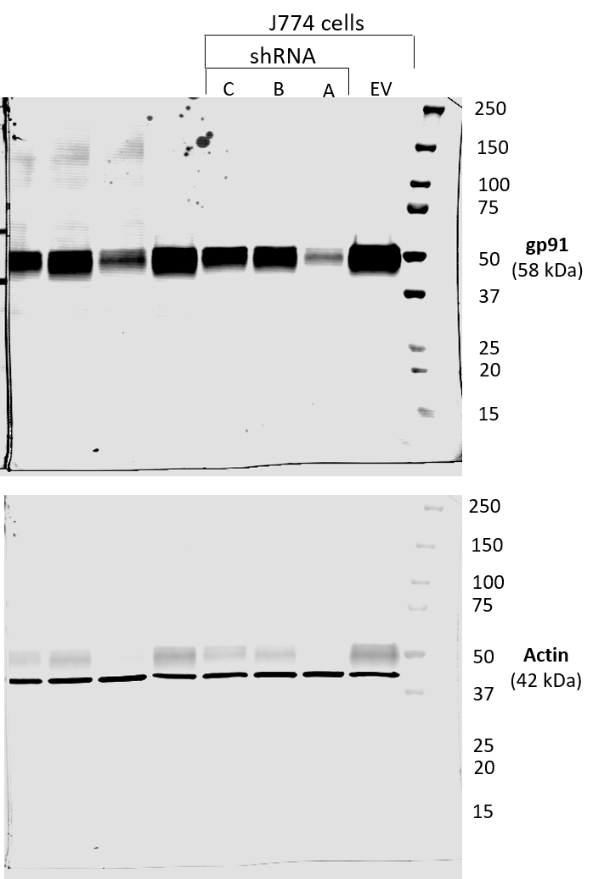
**Full Blot from SF 1B (gp91) Full Blot from SF 1B (p22)**


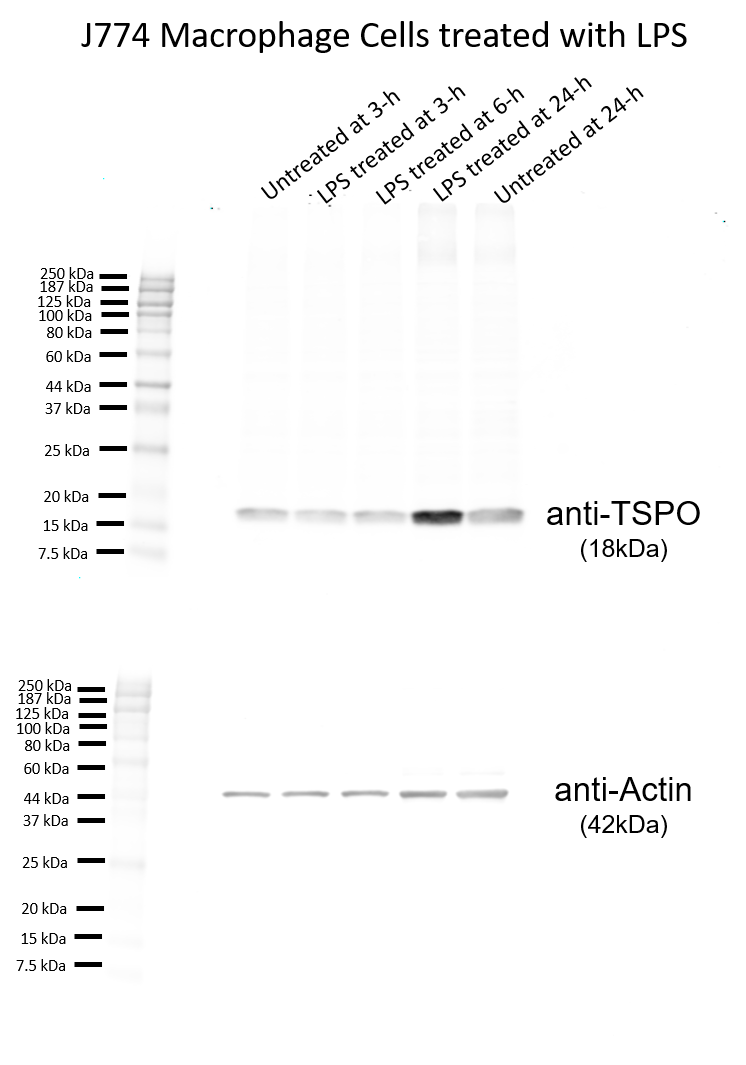
**Full Blot from SF 1C**

**
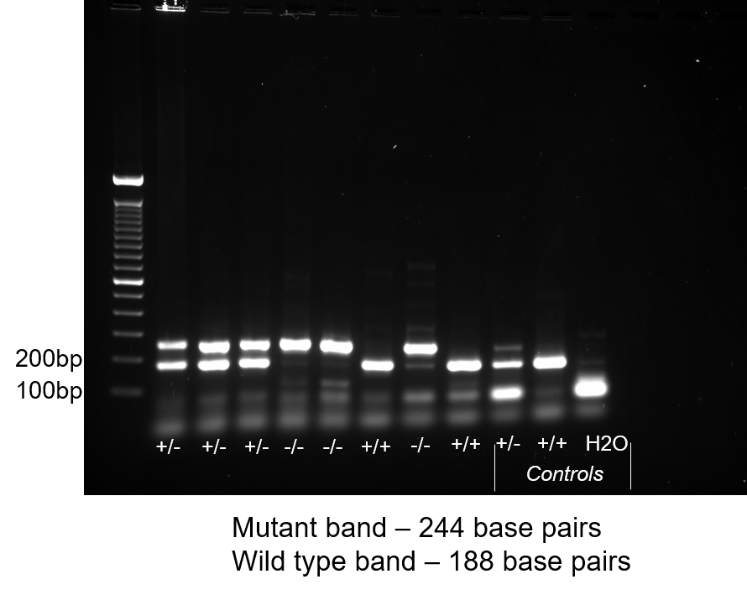
Full Blot from SF 1D(i)**


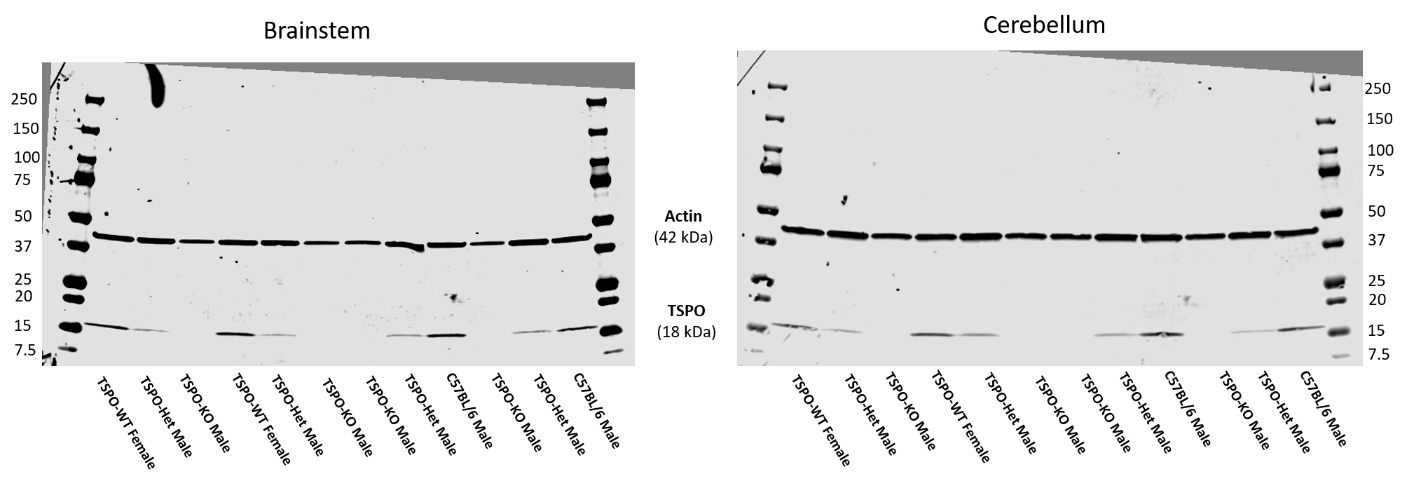
**Full Blot from SF 1D(iii)**

**Supplemental Figure S1 (continued):** Full blots of data presented in Supplemental Figure S1.


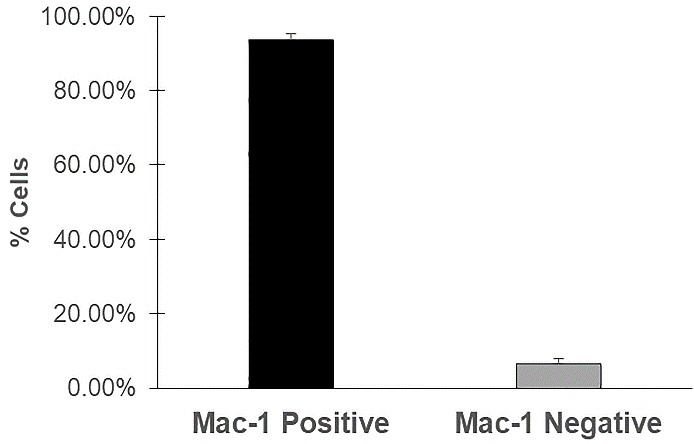


**A.**


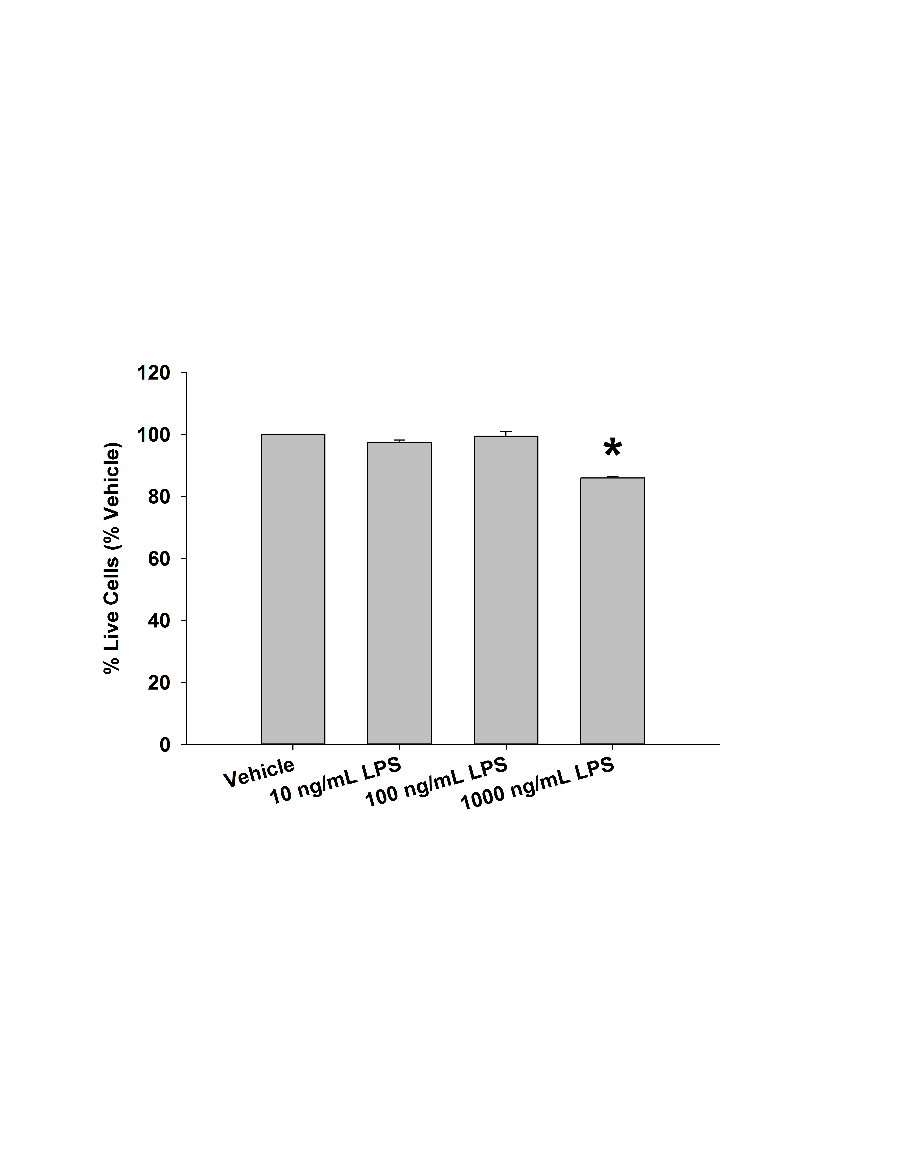


**B.**

**Supplemental Figure S2:** Purity and lipopolysaccharide (LPS) cytotoxicity in primary microglia cultures. (A) Purity of cultures was assessed via microglia Mac-1 immunostaining. Of the cells extracted from mixed glial cultures via shaking, 93.6% of cells were positive for Mac-1, with 6.4% of cells being positive for DAPI only. (B) Primary murine microglia were dosed with specified doses of LPS for 18 hours and the percentage of live and dead cells were counted via the Live/Dead assay. Increased cytoxicity was seen with 1 ug/mL of LPS at 18 hours (* = p < 0.001 compared to all other groups; ANOVA: F_3,11_ = 80.407). LPS exposure of 100 ng/ml for 18 hrs was used for all studies. Data are expressed as mean ± s.e.m. n=6 independent experiments for purity measurement; n=3 independent experiments for cytotoxicity.

**Full Blot from Figure 1A and 1D (TSPO) – *same blot as gp91 below***


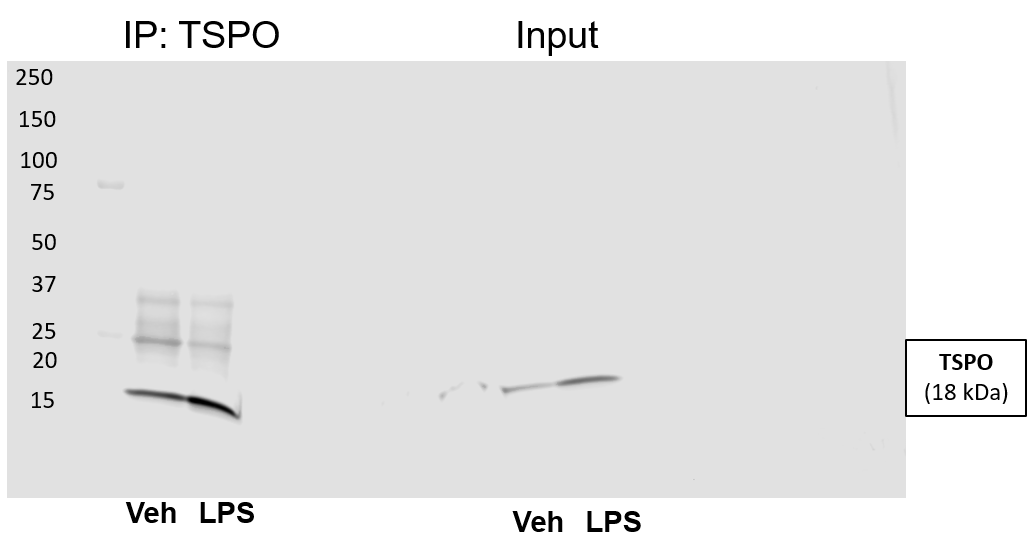


Flow

Through

IP:TSPO

Pellet

Input

Veh LPS

Veh LPS

Veh LPS

Veh LPS

**Quick Detection Kit (high intensity scan)**

**Full Blot from Figure 1A and 1D (gp91) – *same blot as TSPO above***

IP:TSPO

Veh LPS

LPS Veh

Veh LPS

Veh LPS

Pellet

Input

IP:TSPO

Pellet

Input

LPS Veh

Veh LPS

Veh LPS

Veh LPS


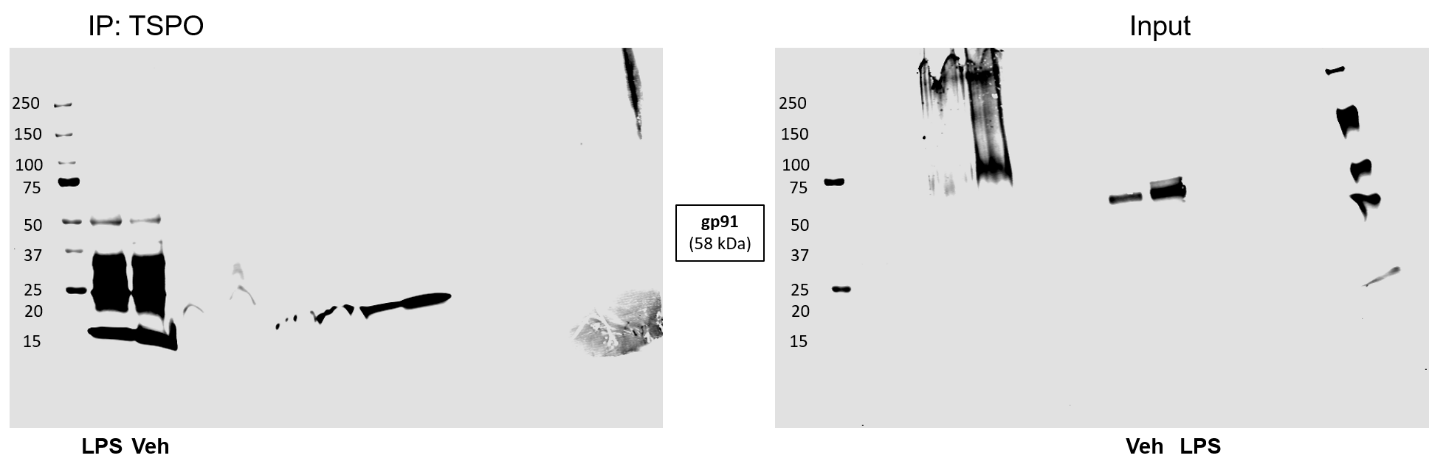


Flow

Through

Flow

Through

**Quick Detection Kit (low intensity scan)**

**Anti-mouse-800 2° Ab (low intensity scan)**

**Full Blot from Figure 1A and 1D (p22) – *same blot as VDAC below***


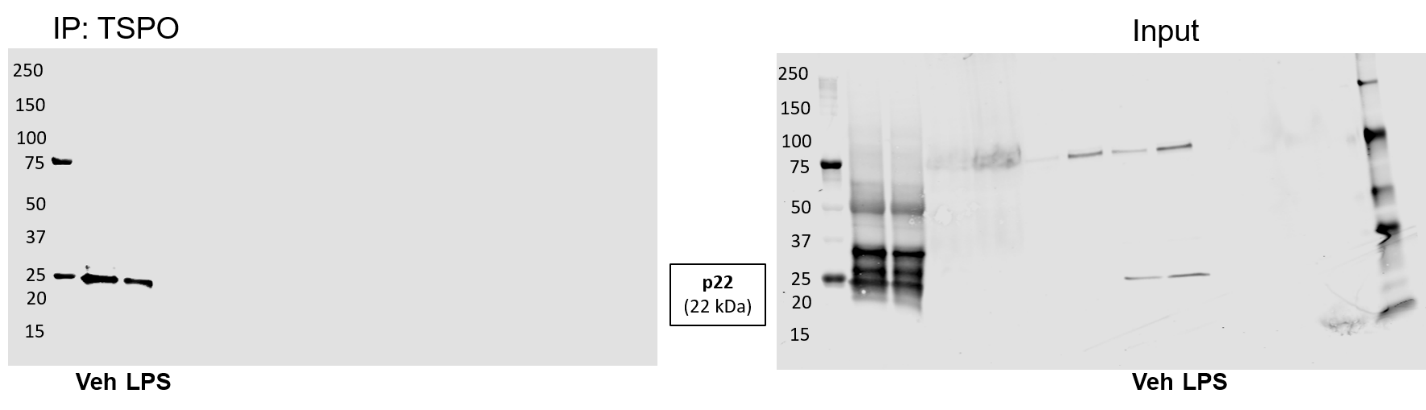


Veh LPS

Veh LPS

Veh LPS

Veh LPS

Input

Pellet

IP:TSPO

Input

Pellet

IP:TSPO

Veh LPS

Veh LPS

Veh LPS

Veh LPS

Flow

Through

Flow

Through

**Quick Detection Kit (low intensity scan)**

**Anti-mouse-800 2° Ab (high intensity scan)**

**Full Blot from Figure 1A and 1D (VDAC) – *same blot as p22 above***

Veh LPS

Input

Pellet

IP:TSPO


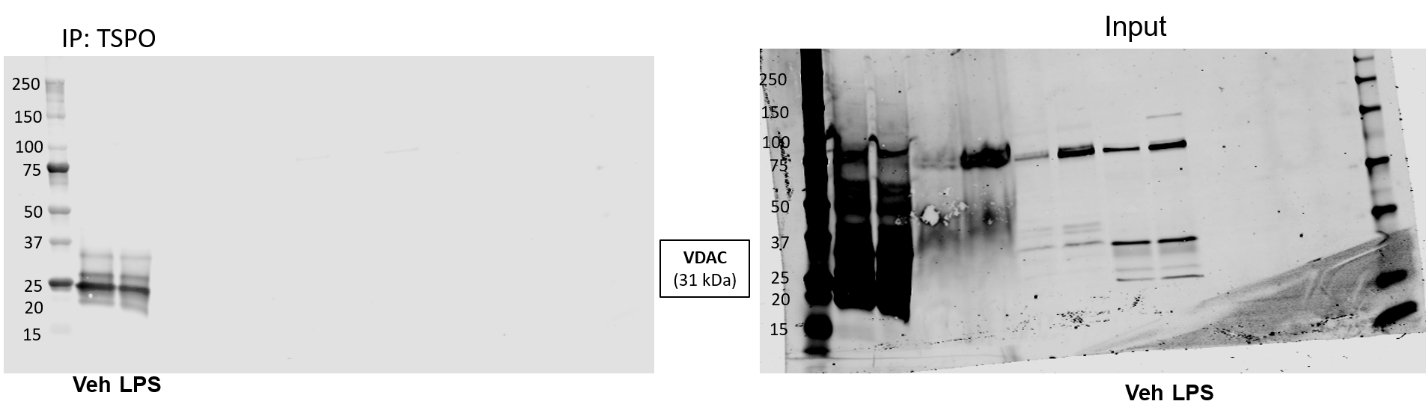


Input

Pellet

IP:TSPO

Veh LPS

Veh LPS

Veh LPS

Veh LPS

Veh LPS

Veh LPS

Veh LPS

Flow

Through

Flow

Through

**Quick Detection Kit (low intensity scan)**

**Quick Detection Kit (high intensity scan)**

**Supplemental Figure S3:** Full blots of data presented in Figure 1.


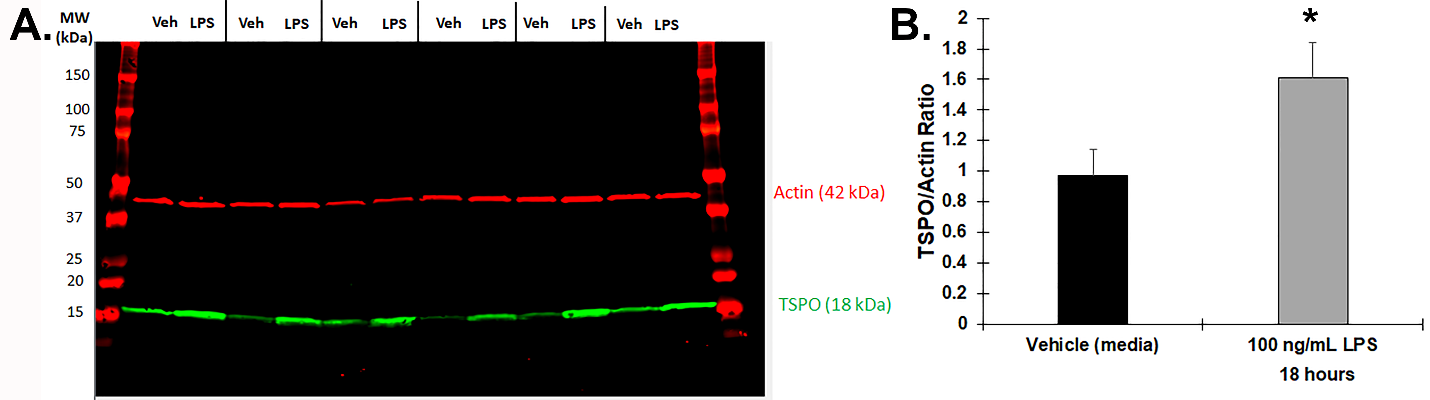


**Supplemental Figure S4:** (A) TSPO western blot of primary microglia treated with media or 100 ng/mL LPS for 18 hours. (B) LPS stimulation significantly increases the expression of TSPO. Each lane contained 25 ug of protein. Protein from six (6) independent experiments were run on the same gel. To correct for loading, data are expressed as TSPO/Actin Ratio and as mean ± s.e.m. Paired t-test: *p=0.0006 compared to vehicle


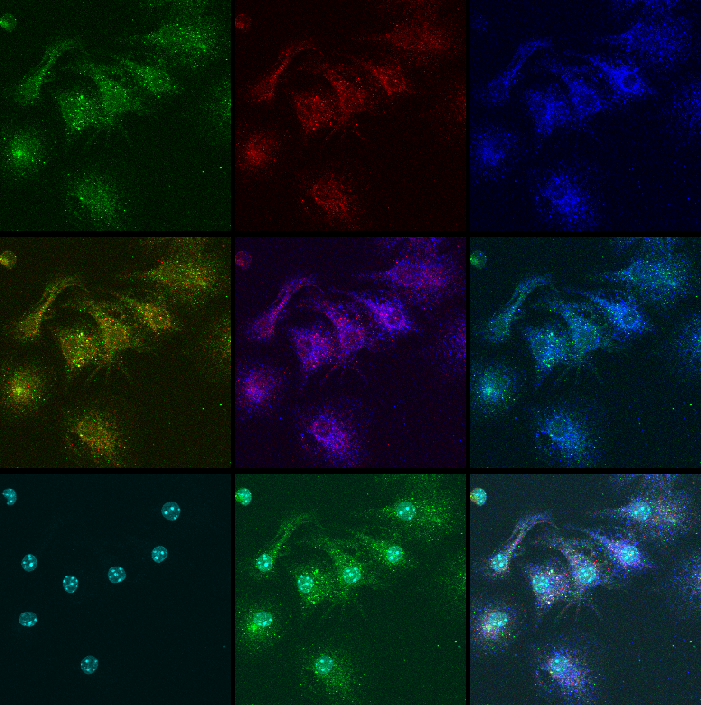


TSPO

VDAC

gp91

TSPO/VDAC

VDAC/gp91

TSPO/gp91

DAPI

DAPI/TSPO

Merged


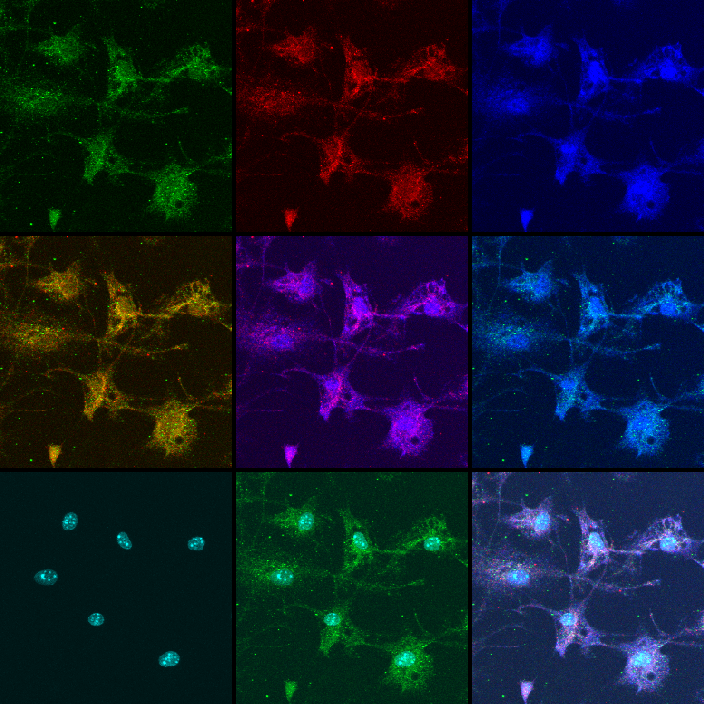


TSPO

VDAC

p22

TSPO/VDAC

VDAC/p22

TSPO/p22

DAPI

DAPI/TSPO

Merged

**A.**

**B.**

**Supplemental Figure S5:** TSPO/gp91/VDAC and TSPO/p22/VDAC immunolabeling in primary microglia. Representative triple labeled immunofluorescent confocal images of microglia used for analyses represented in Figure 2. Imaging and analyses confirmed that TSPO colocalized with the mitochondrial protein VDAC, as represented by the yellow color, and that TSPO colocalized with both gp91phox and p22phox as represented by the purple/magenta colors. Images were taken with a 60x objective with a 1.6x zoom.

**
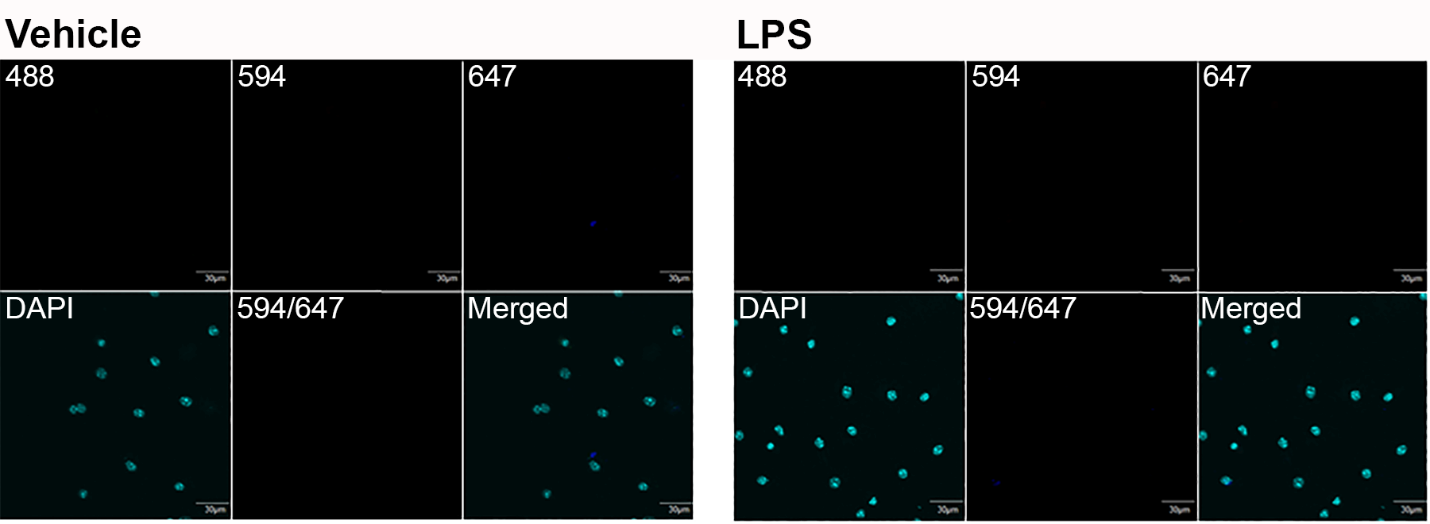
A**.


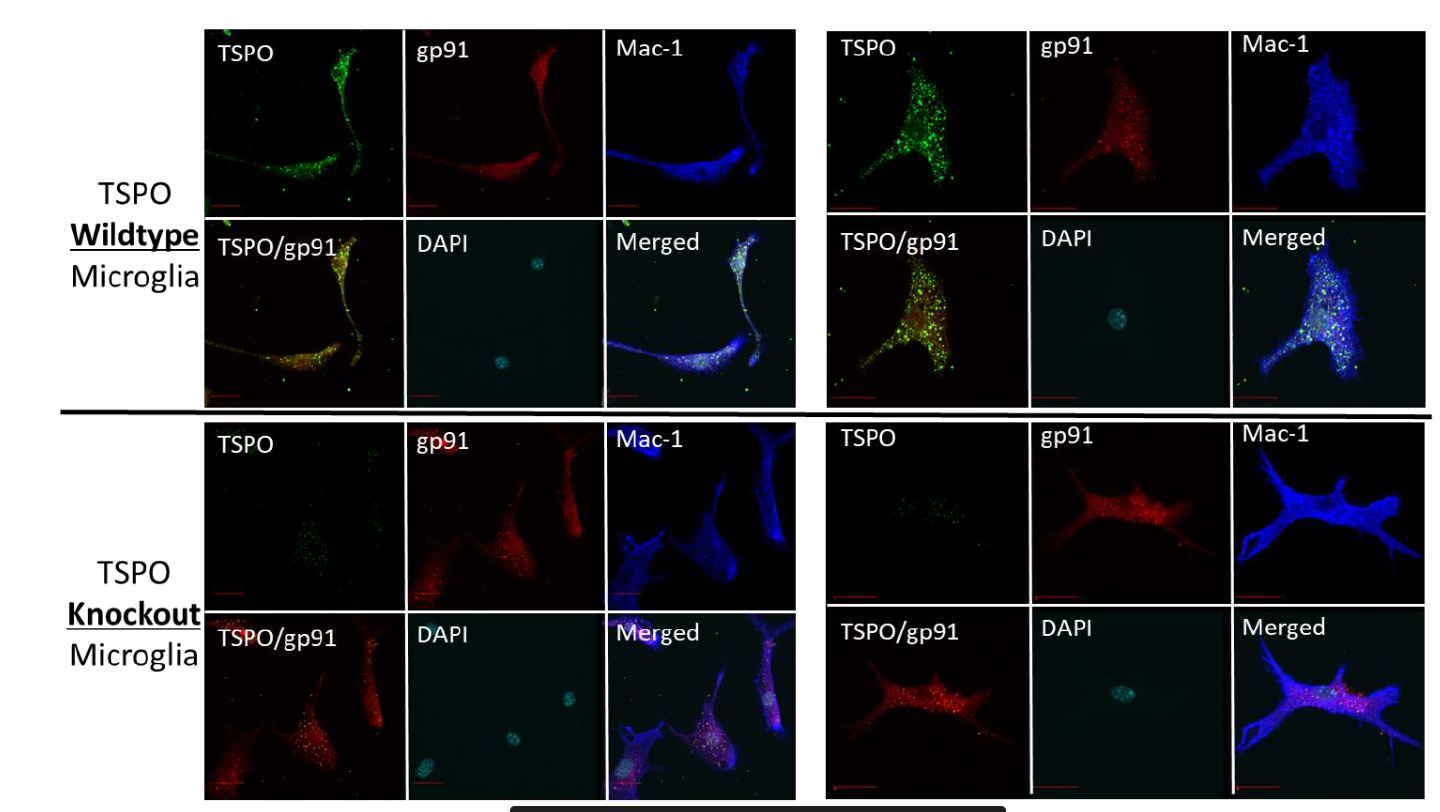
**B.**

**Supplemental Figure S6: A.** Negative control (absence of primary antibody) confocal images of primary microglia exposed to vehicle and LPS (100 ng/mL for 18 hours) for immunocytochemistry experiments presented in Figure 2. B. Confocal imaging of TSPO-WT and TSPO-KO microglia confirms the deletion of TSPO.


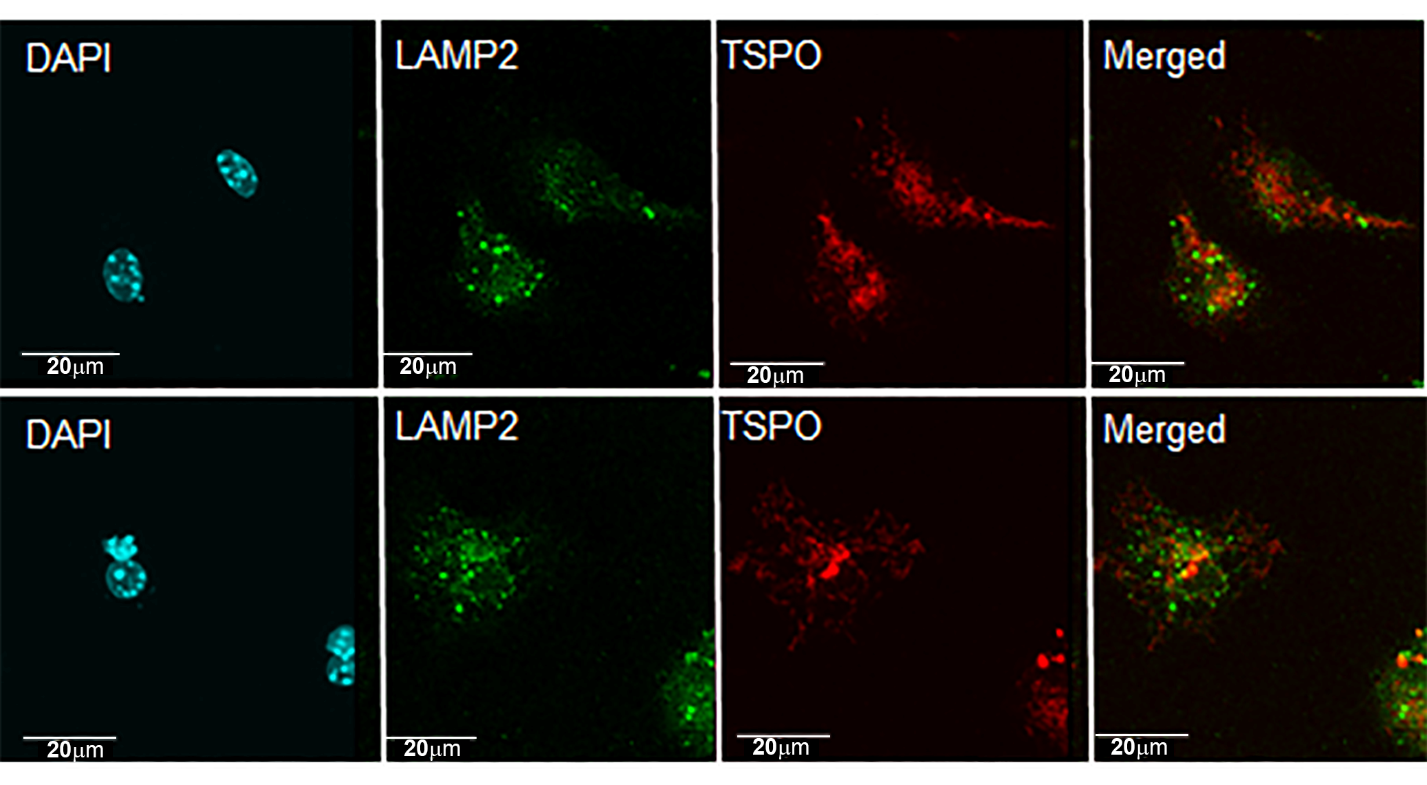


**Supplemental Figure S7:** TSPO/LAMP2 immunolabeling in primary microglia. Representative double label immunofluorescent confocal images of microglia used for analyses represented in Figure 2. Imaging and analyses confirmed that TSPO has low level of colocalization with the lysosomal marker, LAMP-2 (Vehicle = 20.13% ± 7.25%). n= 3 independent experiments for TSPO/LAMP2 labeling with > 35 microglia counted per treatment condition per experiment. Scale bar = 20 um.

**Supplemental Table S8:** LPS activation shows no effect on the number of gp91^phox^ and p22^phox^ interactions with TSPO using the Proximity Ligation Assay (PLA) in primary microglia

|  | **TSPO + gp91** | | **TSPO + p22** | | **TSPO + VDAC** | |
| --- | --- | --- | --- | --- | --- | --- |
|  | **Vehicle** | **LPS** | **Vehicle** | **LPS** | **Vehicle** | **LPS** |
| **TSPO + protein** | 4.5 ± 1.3 | 4.7 ± 1.6 | 5.6 ± 1.2 | 6.5 ± 1.5 | 9.2 ± 2.2 | 7.3 ± 2.0 |
| **TSPO only** | 1.3 ± 0.9 | 0.7 ± 0.3 | 0.9 ± 0.4 | 1.0 ± 0.4 | 0.7 ± 0.3 | 0.7 ± 0.1 |
| **Protein only** | 1.1 ± 0.4 | 1.1 ± 0.1 | 1.3 ± 0.2 | 2.4 ± 0.4 | 1.4 ± 0.4 | 1.2 ± 0.2 |
| **No primary** | 0.8 ± 0.3 | 0.8 ± 0.3 | 0.6 ± 0.3 | 0.4 ± 0.2 | 1.7 ± 0.6 | 1.1 ± 0.2 |

Vehicle data presented are the same data graphed in Figure 4.

Data are expressed as mean ± s.e.m. n = 6-7 independent experiments with 30+ cells counted per treatment and per labeling condition.


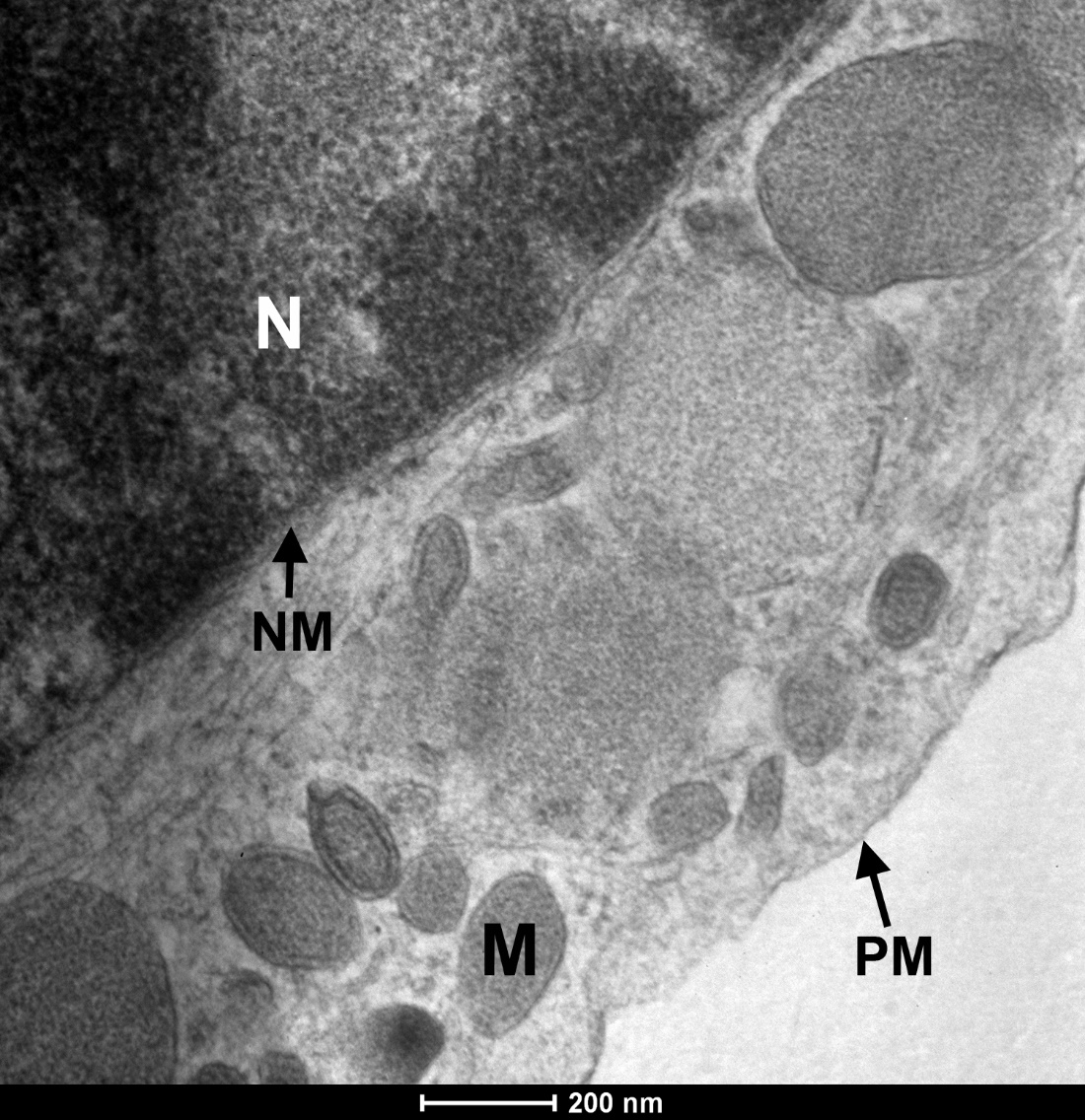


**Supplemental Figure S9:** Negative control (absence of primary antibody) for immunogold electron microscopy of TSPO in primary microglia presented in Figure 4. N = nucleus; NM = nuclear membrane; M = mitochondria; PM = plasma membrane


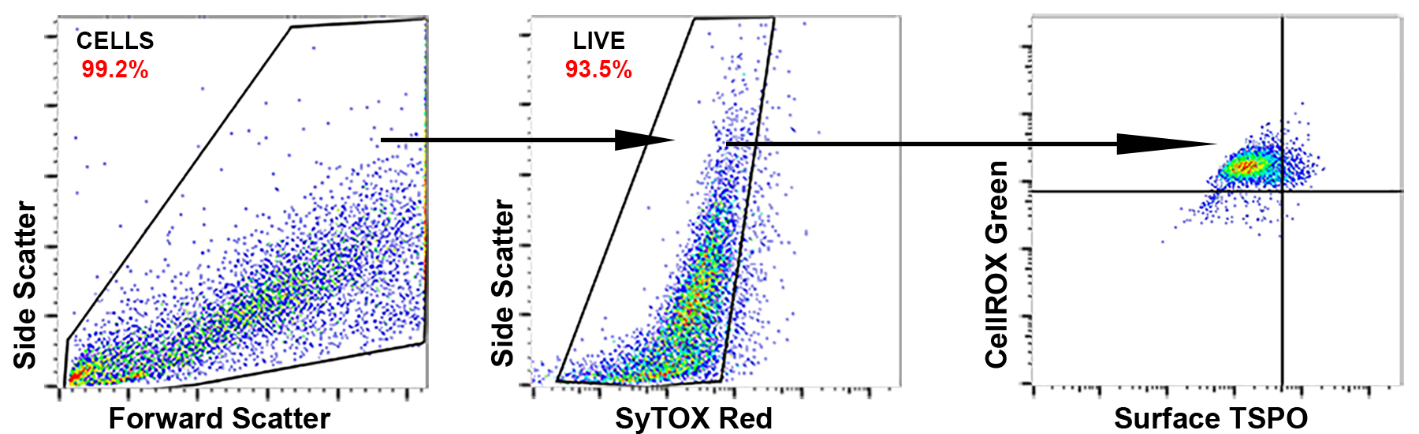


**Supplemental Figure S10:** Representative gating strategy for live primary microglia by excluding SYTOX^®^ positive cells.


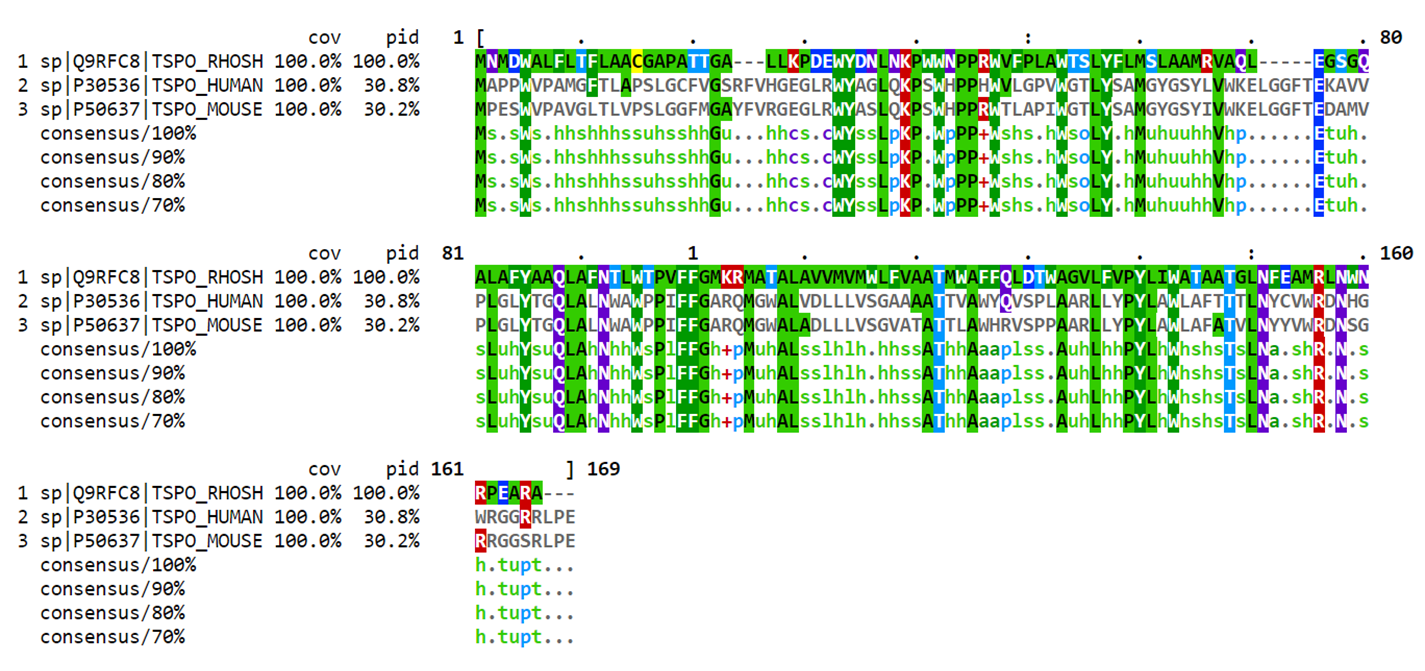


**Supplemental Figure S11:** Alignment of TSPO protein sequences. The primary sequences of human (hTSPO), mouse TSPO (mTSPO) and *Rhodobacter sphaeroides* TSPO (rTSPO) were performed using the Multiple Alignment Viewer, MView, from the European Bioinformatics Institute (EMBL-EBI).
